# Development and Characterization of Liposome-Based Dermocosmetic Formulations with Red Grape Pomace and *Polygonum cuspidatum* Extracts

**DOI:** 10.3390/antiox14101182

**Published:** 2025-09-28

**Authors:** Cristiana Radulescu, Radu Lucian Olteanu, Claudia Lavinia Buruleanu, Raluca Maria Stirbescu, Andreea Laura Banica, Ramona-Daniela Pavaloiu, Fawzia Sha’at, Maria Monica Petrescu, Gabriela Stanciu

**Affiliations:** 1 Faculty of Sciences and Arts, Valahia University of Targoviste, 13 Sinaia Alley, 130004 Targoviste, Romania; cristiana.radulescu@valahia.ro; 2 Doctoral School Chemical Engineering and Biotechnology, National University of Science and Technology Politehnica of Bucharest, 313 Splaiul Independenței, 060042 Bucharest, Romania; banica.andreea@icstm.ro; 3 Academy of Romanian Scientists, 3 Ilfov, 050044 Bucharest, Romania; 4 Faculty of Environmental Engineering and Food Science, Valahia University of Targoviste, 13 Sinaia Alley, 130004 Targoviste, Romania; lavinia.buruleanu@valahia.ro; 5 Institute of Multidisciplinary Research for Science and Technology, Valahia University of Targoviste, 13 Sinaia Alley, 130004 Targoviste, Romania; stirbescu.raluca@icstm.ro; 6 National Institute for Chemical-Pharmaceutical Research and Development, ICCF, Synthesis of Bioactive Substances and Pharmaceutical Technologies Department, 112 Vitan Avenue, 3rd District, 031299 Bucharest, Romania; fawzya.shaat@gmail.com (F.S.); maria.m.petrescu@gmail.com (M.M.P.); 7 Department of Chemistry and Chemical Engineering, Faculty of Applied Sciences and Engineering, Ovidius University of Constanta, 900527 Constanta, Romania; gstanciu@univ-ovidius.ro

**Keywords:** grape pomace extract, Japanese knotweed extract, antioxidant activity, phenolic compounds, HPLC/FLD/DAD, encapsulation, dermatocosmetic gels, liposome-loaded

## Abstract

The use of antioxidants in the dermatocosmetic industry has become increasingly popular to help protect and stabilize other sensitive active ingredients, prolonging the effectiveness and durability of the cosmetic product. Grape pomace, as the main by-product generated through winemaking, and *Polygonum cuspidatum*, concentrate bioactive metabolites with high antioxidant activity. Hydroalcoholic extracts obtained from grape pomace (Merlot and Feteasca Neagra varieties) and the root and flower of Japanese knotweed, respectively, alone and in mixtures, were characterized, and preliminary assays were conducted for their incorporation in two gel-based cosmetic formulations. The characterization of the extracts revealed the presence of catechin, vanillic acid, caffeic acid, myricetin, resveratrol, and kaempferol. The hydroalcoholic extract of *P. cuspidatum* flower and root was found to have the highest content of total phenolic compounds (10.920 ± 0.268 mg GAE/mL, respectively, 4.751 ± 0.072 mg GAE/mL), and the highest antioxidant activity (expressed as DPPH Radical Scavenging Capacity, IC_50_) by 28.04 ± 1.12 µg GAE/mL and 83.91 ± 1.13 µg GAE/mL, respectively. Catechin was the most abundant polyphenol found in pomace extract (687.87 mg/kg). The type and the concentration of the plant extract used in dermatocosmetic gel formulations influenced their antioxidant activity. Encapsulation of *P. cuspidatum* flower extract in liposomes prior to their incorporation into the gel formulation demonstrated the role of liposomes in enhancing the stability and modulation of phenolic compound delivery. It is worth noting that this dermatocosmetic formulation, which contains the flower extract of *P. cuspidatum*, was the subject of a pending patent application.

## 1. Introduction

The dermatocosmetic industry is constantly evolving, offering new methods and products to improve the texture and overall appearance of skin. The effectiveness of a cosmetic product, whether it is a dermatocosmetic or a regular one, depends on its formula, the concentration of active ingredients, and the way it is used. In certain situations, some regular cosmetics can be as effective or even more effective than dermatocosmetics. While dermatocosmetics are often marketed as superior due to their clinical backing and targeted formulations, there are several reasons why regular cosmetics can sometimes match, or even outperform them, in specific situations such as (*i*) ingredient innovation in regular cosmetics; (*ii*) comparable hydration and antiaging effects; (*iii*) gentler formulations for sensitive skin; (*iv*) user experience and consistency [1,2,3,4]. Considering the benefits of dermatocosmetics, it is beneficial to know that they are designed to combine cosmetic and therapeutic benefits, aiming to have a positive impact on the skin. Choosing the right one depends on several factors, including skin type, existing problems, and individual care goals [5]. In addition to their therapeutic effects, dermatocosmetics are also used for aesthetic benefits through innovative formulations containing active ingredients that can penetrate the upper skin layers [5]. These products, developed through extensive research, are dermatologically tested to ensure a high level of safety and efficacy in treating various conditions. In addition to their therapeutic effects, dermatocosmetics are also used for aesthetic benefits, thanks to innovative formulations that incorporate active ingredients that can penetrate the upper layers of the skin. These products, created following extensive research, are dermatologically tested to give them a high level of safety and efficacy in treating various conditions (often specified with labels/inscriptions such as ‘Free parabens’, ‘Free microplastics’, ‘Natural extract’, ‘Not tested on Animals’, ‘Animal-Friendly Cosmetic’ or ‘Vegan’, ‘Cruelty-Free’, and so on), but also to increase consumer confidence in these products [6,7,8,9]. Article 20 from the EU Cosmetic Products Regulation [10] mentions in product claims the following aspects: (*i*) prohibition of misleading claims; (*ii*) truthfulness; (*iii*) evidence-based; (*iv*) fairness. Based on these consumer concerns, worldwide efforts have been made to find the safest and most reliable dermatocosmetic products for people to use at the recommendation of specialists (i.e., dermatologists, pharmacists, cosmetologists) in compliance with requirements provided by Registration, Evaluation, Authorisation and Restriction of Chemicals (REACH) and the Cosmetic Products Regulation [9,10,11]. In this regard, the use of natural plant or fruit extracts, rich in antioxidants, brings added value and trust to the cosmetics market [12]. On the other hand, extracts from residues resulting from the fermentation of fruits or other vegetables can serve as a way to valorize these residues, aligning with the principles of the circular economy (the 7 R’s: Reuse, Reduce, Recycle, Repurpose, Repair, Recover, Renovate).

In previous research, the authors have demonstrated that grape pomace is an excellent raw material for extracting active compounds with added value for cosmetics and health foods [6,13,14]. A series of research has been the subject of patents or patent applications [14,15] as well. Based on the phytochemical screening and data correlation of various grapes and pomace grape extracts investigated in the last six years [6,13,14,15], the authors decided that a mixture of Merlot, a consecrated variety worldwide, and Feteasca Neagra, an autochthonous variety with an amazing ancient flavor (from the Dacian period), can be an alternative to obtain a complex mixture extract, who can be used in different cosmetic formulation.

*P. cuspidatum* is a traditional perennial medicinal plant from the *Polygonum* family, commonly found in China and Japan. It is viewed as invasive in regions outside of Asia due to the aggressive growth of its rhizomes. In traditional medicine, *P. cuspidatum* is utilized in the form of powders, decoctions, or infusions to treat infections, inflammatory diseases, jaundice, skin burns, hyperlipidemia, chronic bronchitis, hypertension, and high cholesterol levels [16]. The therapeutic properties of the *P. cuspidatum* plant, commonly known as Japanese knotweed, are attributed to its unique phytochemical composition. Considering the customer’s preference for natural products, especially in the field of dermatocosmetics for both treatment and beauty, the cosmetics and pharmaceutical industries are introducing a variety of sustainable alternatives for their production. These natural and safe products utilize innovative techniques, including encapsulation, microencapsulation, and liposome-based delivery systems, to improve the stability and bioavailability of sensitive active compounds [17,18,19]. Liposomal encapsulation has emerged as a particularly promising strategy in dermatocosmetic science due to its unique structural and functional properties. Liposomes are spherical vesicles composed of one or more phospholipid bilayers that can encapsulate both hydrophilic and lipophilic substances. Their biocompatibility and structural similarity to biological membranes make them ideal carriers for topical applications. In the context of polyphenol-rich extracts, liposomal encapsulation offers several critical advantages [17]. Firstly, it provides a protective barrier against environmental stressors such as oxidation, UV radiation, and temperature fluctuations, thereby preserving the stability and functional integrity of sensitive bioactives like polyphenols [19,20]. Secondly, the nanoscale size and amphiphilic nature of liposomes enhance the penetration of active compounds through the stratum corneum and into deeper layers of the skin, which is often a limitation in conventional formulations [17]. Additionally, liposomes allow for a controlled and sustained release of active ingredients over time, which may prolong their biological effects and reduce the need for frequent application. This consistent delivery is especially important in dermatocosmetic products focused on antioxidant protection, skin renewal, and anti-aging, where ongoing exposure to active compounds can greatly enhance effectiveness [20]. Given these advantages, liposomal systems have gained widespread attention in the formulation of advanced skincare products, offering a scientifically sound approach to improving both the performance and user experience of natural ingredient-based cosmetics.

Considering the previously mentioned aspects, this study explores a topic of interest to consumers: the development of new dermatocosmetic products enhanced with natural extracts from plants or other by-products that have been subjected to a thorough phytochemical screening. Red grape pomace extracts, such as Feteasca Neagra and Merlot, have been extensively characterized based on research by Radulescu et al. [6,13,14]. Although there is comprehensive information on the phytochemical profile of Japanese knotweed root extracts, the chemical composition of knotweed flower extracts remains incomplete and unclear. The uniqueness of this study comes from two key aspects. The first objective of this study was to characterize mixtures of red grape pomace from Feteasca Neagra and Merlot varieties, as well as extracts from the roots and flowers of Japanese knotweed. The second objective was to investigate the phytochemical profile of complex mixtures that combine the pomace with the root and flower extracts of knotweed through statistical analysis. Finally, the third objective was to incorporate the knotweed flower extract into liposomes for various dermatocosmetic formulations. To improve the stability, bioavailability, and dermal delivery of knotweed flower extract rich in polyphenolic compounds, this was first encapsulated in liposomes before being added to the gel formulation. In the current study, the choice to use either a 1.0% or 1.25% Carbopol gel matrix was intentional, focusing on the viscosity, texture, and structural stability of the obtained cosmetic formulations. These concentrations ensure that the liposomes are evenly dispersed while maintaining a skin-compatible pH. This approach supports both the functional and sensory properties of the obtained final dermatocosmetic products.

## 2. Materials and Methods

### 2.1. Materials and Reagents

Grape pomace was harvested at SC AMB WINE COMPANY SRL, Batos vineyards, Transylvania region, Romania. The red grape pomace (i.e., Feteasca Neagra and Merlot varieties) was collected after fermentation initiation and pressing in autumn 2024, divided into two fractions: stalks and a mixture of grape seeds and skins. It was used semi-dried during the extraction process. Meanwhile, *P. cuspidatum*, a perennial plant, was collected around the village of Batos in the Transylvania region of Romania using sterile, disposable instruments and materials. The Japanese knotweed was dried at 20 °C, and then the flower and root were separated. Both the grape pomace, i.e., Merlot and Feteasca Neagra varieties, and the Japanese knotweed, in the form of root and flower extracts, were coded; therefore, the results were reported using the assigned codes (Table 1).

All reagents (mainly from Merck KGaA, Darmstadt, Germany, Carl Roth, Karlsruhe, Germany, and Biosolve Chimie SARL, France) were of HPLC grade, such as: hydrochloric acid, HCl 37% (ACS.ISO.Reag.Ph.Eur, 1.19 kg/L); ethyl alcohol, C_2_H_5_OH p.a., min. 99% (*v*/*v*), M = 46.07 g/mol; methyl alcohol, CH_3_OH, 0.79 kg/L, M = 32.04 g/mol; vanillin C_8_H_8_O_3_, M = 152.14 g/mol; (+)-catechin C_15_H_14_O_6_, M = 290.28 g/mol; quercetin dihydrate, C_15_H_14_O_9_, 3,3′,4′,5,7-pentahydroxyflavone, p.a., min. 95%, M = 302.24 g/mol; anhydrous gallic acid, C_7_H_6_O_5_, 3,4,5-acid trihydroxybenzoic, p.a. ACS, min. 98%, M = 170.12 g/mol; Folin–Ciocalteu reagent; TRIS H_2_NC(CH_2_OH)_3_, 2-amino-2-(hydroxymethyl)propane-1,3-diol, p.a., M = 121.14 g/mol, ACS.ISO.Reag.Ph. Eur; acetonitrile, CH_3_CN, M = 41.05 g/L; vanillic acid, C_8_O_4_H_8_, 97.0%, M = 168.15 g/mol; caffeic acid, C_9_H_8_O_4_, M = 180.16 g/mol; myricetin, C_15_H_10_O_8_, 96.0%, M = 318.24 g/mol; resveratrol, C_14_H_12_O, 99%, M = 228.24 g/mol; kaempherol, C_15_H_10_O_6_, 97%, M = 286.24 g/mol; Carbopol 940; glycerin; 2,2-diphenyl-1-picrylhydrazyl (Tokyo Chemical Industry, Tokyo, Japan); lavandin essential oil (Fares, Orastie, Hunedoara, Romania); potassium chloride, KCl, p.a., min. 99.8%, sodium acetate trihydrate, CH_3_COONa·3H_2_O, p.a., min. 98.5%, sodium carbonate Na_2_CO_3_, p.a., min.96%, and aluminum chloride hexahydrate, AlCl_3_·6H_2_O, p.a., min. 96% (Chimopar SA, Bucharest, Romania); sodium hydroxide, NaOH, p.a., min. 99.02% and sodium nitrite, NaNO_2_, p.a., 99.5%(Lach-Ner s.r.o. Neratovice, Czech Republic). Deionized water (i.e., conductivity below 0.5 µS/cm at 25 °C) was used for the dilution and sample preparation.

### 2.2. Countercurrent Extraction Under Pressure and Concentration of Samples

The extracts of pomace and *P. cuspidatum* (root and flowers) were obtained in a pilot plant owned by the company SC NIRVANA SRL. The plant consists of two systems: one for countercurrent extraction under 8 bar pressures, and one for extract concentration. The extraction system is designed for the extraction of bioactive components from fresh or dehydrated vegetable raw materials, in liquid solvents (water, alcohol, glycerin, propylene glycol, oil, vinegar, etc.). The extraction process was carried out at room temperature, at pressures of maximum 8 bars, under complete microprocessor control of all equipment functions (double pressure/depression effect, double washing system, possibility of storing and recording 50 different extraction programs, real-time visual display of process parameters and sequences of each cycle, alarm system, etc.), in a stainless steel AISI304 external vessel of 300 L capacity, with heating for solvent recirculation. The second step is the concentration of heat-sensitive extracts using a professional system equipped with a tank (extraction chamber) with a capacity of 300 L in AISI304 stainless steel reinforced and insulated in such a way as to limit heat losses and reduce energy consumption; innovative rapid heating system for energy saving, high efficiency vacuum pump for regulating the vacuum in the system and steam cooling circuit through closed circuit cooler. Extraction was achieved using 70% ethanol as the extraction solvent, at a ratio of plant material: extraction solvent equal to 1:2 (*m*/*v*) and 1:1 (*m*/*v*) for pomace and *P. cuspidatum*, respectively. Extraction time was set at 25 cycles × 14 min.

### 2.3. Total Condensed Tannins

The total condensed tannins (TCT) was determined using the procedure proposed by Broadhurst and Jones [21] based on the reaction of flavanols with vanillin in acidic media to form a colored compound that absorbs at 500 nm and gives the solution a red coloration that is quantified calorimetrically [21,22]. The extract samples were centrifuged and filtered before use. The procedure detailed in a previously published paper [13] involved: (1) adding of 3 mL of 2.4% vanillin solution (in methanol 60%) at 0.5 mL sample (extract) and homogenizing (vortex) the mixture; (2) 1.5 mL of 30% hydrochloric acid was added to the mixture and homogenized again (vortex); (3) maintaining the mixture for 15 min at 20 ± 2 °C, then for 13 min at 25 °C. Blank and interference correction samples (anthocyanins) were made similarly, using 0.5 mL of distilled water instead of the extract and 3 mL of methanol instead of the vanillin solution, respectively. The sample absorbance measured at 500 nm, using an Evolution 260 Bio UV-Visible spectrophotometer (Thermo Fisher Scientific Inc., Madison, WI, USA), was calculated considering the contributions of the absorbance of the blank and the interference correction sample. The TCT content was determined based on the absorbance of the sample and the calibration curve, i.e., (+)− catechin, and expressed in catechin equivalents (CE) as mg CE/mL [13].

### 2.4. Total Anthocyanins Content

The total anthocyanin content (TAC) of grape pomace, Japanese knotweed, and grape pomace—Japanese knotweed extracts were determined using the pH variation method proposed by Giusti and Wrolstad [23]. The procedure involved the following steps [13]: (1) setting the dilution factor (DF) of each sample by using a KCl 0.025 M buffer solution (pH = 1.0) as the absorbance of the sample was less than 1.2; DF was set as the final volume of the sample divided by the initial volume of the extract; (2) recording the sample spectrum (260–800 nm; distilled water blank) to verify the position (λ_max_) and the amplitude (maximum absorbance value) of the peak; (3) two dilutions of each extract sample were prepared, one with KCl 0.025 M buffer solution (pH = 1.0) and the second with CH_3_COONa 0.4 M buffer solution (pH = 4.5) and were maintained in equilibrium for 15 min; (4) the absorbance for the two sample dilutions was measured at 700 nm (*A*_700nm_) and the one corresponding to the maximum absorption (*A*_520nm_); (5) the absorbance of the diluted sample was calculated according to Equation (1):

(1) $$A={\left(A_{520nm}-A_{700nm}\right)}_{pH=1.0}-{\left(A_{520nm}-A_{700nm}\right)}_{pH=4.5}$$

The total anthocyanins content (TAC) was quantified as the concentration of monomeric anthocyanin pigment (MAP), expressed as mg/L, and calculated with Equation (2):

(2) $$MAP=\frac{A\cdot MW\cdot DF\cdot 1000}{\varepsilon \cdot l}$$

where *A* is the absorbance of the diluted sample; *MW* is the molecular mass (g/mol); *DF* is the dilution factor; ε represents the molar absorptivity expressed as L/(mol·cm); *l* is the optical path length (1 cm). According to the method, if *ε* for the majority pigment is not available, or if the composition of the sample is unknown, the pigment content must be calculated as cyanidin-3-glucoside, where *MW* = 449.2 g/mol and *ε* = 26,900 L/(mol·cm) [24,25].

### 2.5. Total Flavonoids Content

The total flavonoids content (TFC) was measured using the aluminum chloride assay, which is based on the reaction between flavonoids and aluminum chloride in a weak basic medium, resulting in a yellow-orange complex quantified colorimetrically at 510 nm [22,26]. The procedure [13] involved the following steps: (1) at 1 mL of extract sample was added 4 mL of distilled water and 0.3 mL solution of 5% NaNO_2_; (2) the mixture was stirred (vortex) and allowed to rest for 5 min; (3) 0.3 mL of 10% AlCl_3_ solution was added and the mixture was stirred (vortex) and maintained at rest for 5 min; (4) 2 mL of 1 M NaOH solution was added and stirred again < (5) finally the mixture was brought to a final volume of 10 mL by adding 2.4 mL of distilled water and homogenized. The blank was prepared similarly, using 1 mL of distilled water instead of the extract sample. Absorbance of the sample was measured at 510 nm, using an Evolution 260 Bio UV-Visible spectrophotometer (Thermo Fisher Scientific Inc., Madison, WI, USA), and the total flavonoid content (TFC) was determined based on the calibration curve of quercetin (Sigma Aldrich, Merck KGaA, Darmstadt, Germany). The results were expressed as mg QE/mL (mg quercetin equivalents/mL extract).

### 2.6. Total Polyphenols Content

The total polyphenols content (TPC) was determined according to a standardized procedure [27] with some modifications for optimizing reagent consumption [5,13] using Folin–Ciocalteu reagent in basic medium. The procedure involved the following steps: (1) 1 mL of extract sample was diluted in a volumetric flask with distilled water; determinations were made for a dilution factor (FD) of 50, 100 and 200 to fit the sample absorbance into the linearity range of the gallic acid (GA) calibration curve; (2) 0.5 mL of diluted extract sample was taken into a brown-walled test tube; (3) 2.5 mL of 10% Folin–Ciocalteu reagent were added and the mixture was shaken intermittently (vortex) for 8 min; (4) 2 mL of 8% Na_2_CO_3_ solution were added and the mixture was vortexed; a blank sample was prepared similarly using 0.5 mL of distilled water; (5) the sample and the blank were kept at room temperature for 60 min and the absorbance was read at 765 nm. The total polyphenol content (TPC) was determined based on a GA (Carl Roth GmbH and Co. KG, Karlsruhe-Mühlburg, Germany) calibration curve. The results were expressed as mg GAE/mL (mg gallic acid equivalents/mL extract).

### 2.7. High-Performance Liquid Chromatography—Fluorescence Detection and Diode Array Detection Assay

The determination of chemical compounds in hydroalcoholic extracts (70%) was performed by high-performance liquid chromatography using a 1260 Infinity II chromatograph (Agilent, Santa Clara, CA, USA) equipped with an autosampler, two detectors, such as a fluorescence detector (FLD), a diode array detector (DAD) and a chromatographic column type Agilent ZORBAX RRHD SB-C18 (Agilent Technologies Inc., Santa Clara, CA, USA) with the next characteristics pore size 80 Å, product size 100 × 2.1 mm, particle size 1.8 μm, the column temperature at 20 °C, according to method proposed by Radulescu et al. [13]. OpenLab CDS 2.7 software (Agilent Technologies Inc., Santa Clara, CA, USA) automatically recorded the data. The limit of quantification (LOQ) values for the used standards (catechin, vanillic acid, caffeic acid, myricetin, resveratrol, and kaempferol) were determined (i.e., 0.015 ng/mL, 0.015 ng/mL, 0.014 ng/mL, 0.003 ng/mL, 0.021 ng/mL, and 0.016 ng/mL, respectively). The stock solution containing six polyphenols at a concentration of 100 ppm was prepared by adding aliquots (50–200 μL, Table S1) from individual stocks, then adjusted to 1000 μL with methanol. Calibration solutions ranging from 0.1 to 5.0 ppm were prepared through successive dilutions from the stock solution. For standard solutions (R^2^ > 0.999), the calibration values ranging from 0.2 to 10.0 ppm (Tables S2–S6) were established. The gradient elution program using solvent A (water) and solvent B (acetonitrile) was set as: 2% B from 0 to 8 min, 17% B from 8 to 14 min, 23% B from 14 to 22 min, and then a linear increase to 95% B at 22 min. The flow rate was set at 0.400 mL/min, and the maximum pressure limit was 800 bars.

### 2.8. DPPH (2,2-Diphenyl-Picryl-Hydrazyl) Radical Scavenging Capacity

The DPPH radical scavenging assay was based on the procedure proposed by Shimamura et al. [28] using the inhibition concentration at 50% (IC_50_) to evaluate DPPH radical scavenging capacity [29] of the investigated extracts. The procedure involved the following steps: (1) preparation of the DPPH solution: 7.89 mg of DPPH was dissolved in a 100 mL volumetric flask using ethanol HPLC grade (0.2 mM DPPH); the solution was kept in the dark for 2 h and 1 mL of DPPH solution was taken in a test tube and 0.2 mL of ethanol and 0.8 mL of 2-amino-2-(hydroxymethyl) propane-1,3-diol (Tris)-HCl buffer solution (pH = 7.4) were added; after homogenization (vortex), the absorbance (λ = 517 nm) was measured using a blank (i.e., a mixture of 1.2 mL ethanol and 0.8 mL Tris-HCl buffer solution, pH = 7.4). If the absorbance was in the range of 1.00 ± 0.05, the DPPH solution was used directly for analysis; if the absorbance exceeded the value of 1.05, correction was made by dilution with ethanol; (2) in a brown-walled test tube, 0.2 mL of extract sample was taken, 0.8 mL of Tris-HCl buffer solution and 1 mL of DPPH solution were added and the mixture was homogenized (vortex) for 10 s and after that kept in the dark for 30 min; for each extract sample, five concentrations (by simple or successive dilution) and a control sample (a mixture of 0.2 mL ethanol, 0.8 mL Tris-HCl buffer solution, and 1 mL DPPH solution) were prepared; (3) the absorbance of the sample (A_sample_) and control sample (A_control_) were measured at 517 nm using a blank made from 1.2 mL ethanol and 0.8 mL Tris-HCl buffer solution. The Inhibition Ratio, IR (%), was calculated with Equation (3) [28]:

(3) $$IR\;\left(\%\right)=\left[\left(A_{control}-A_{sample}\right)/A_{control}\right]\cdot 100.$$

Gallic acid (GA) was used for the calibration curve to obtain the reference value necessary to decrease the initial DPPH absorbance by 50% (IC_50_). The same DPPH solution was used both for the sample analysis and the GA calibration curve. DPPH radical scavenging capacity was expressed as IC_50_ in μg GAE/mL (μg GA equivalents/mL extract) [13].

### 2.9. Formulation of Dermatocosmetic Products

Two gel-based cosmetic formulations (Table 2 and Table 3) enriched with grape pomace (Feteasca Neagra and Merlot varieties) and Japanese knotweed (*P. cuspidatum*) extracts were prepared using two different concentrations of Carbopol 940 (1% and 1.25%). Initially, the polymer was allowed to swell in distilled water for 24 h. Glycerin was incorporated to facilitate uniform dispersion. The gelling process was initiated by adding a 2% sodium hydroxide solution under continuous agitation, resulting in the formation of a stable, homogeneous matrix. Extracts and lavandin essential oil (*Lavandula hybrida* L.) were subsequently introduced under constant stirring to ensure even distribution throughout the gel. Apart from its pleasant fragrance, lavandin essential oil (added at 1% concentration) contributes functional benefits to the formulation (Table 2 and Table 3). It is recognized for its soothing, antiseptic, and restorative effects, often utilized in aromatherapy to promote relaxation and ease muscle tension. Furthermore, its inclusion in skincare is valued for its purifying, regenerative, and anti-aging properties.

#### 2.9.1. Visual Inspection

A small quantity of each formulation was applied onto a glass slide and examined under a magnifying lens (4.5× magnification). Observations were made regarding consistency, color, homogeneity, and fragrance.

#### 2.9.2. pH Measurement

The pH levels of the gels were analyzed using a digital pH meter (Mettler Toledo, Columbus, OH, USA) following thorough homogenization of the samples. Measurements were taken after allowing the reading to stabilize for 30 s. Three separate readings were recorded for each formulation at different points. In cases where the variation between values exceeded ±0.2 pH units, the process was repeated to confirm accuracy.

#### 2.9.3. Spreadability Assessment

Spreadability was determined following a modified Ojeda–Arbussa technique. Approximately 1 g of each sample was placed between two glass plates and left for one minute. A progressive series of weights (ranging from 50 g to 750 g) was applied at one-minute intervals, creating cumulative applied masses of 175 g, 225 g, 275 g, 325 g, 625 g, and 875 g. After each load, the spread diameter was measured, and the spreading area (S_i_) was calculated using Equation (4):

(4) $$S_{i}=d_{i}^{2}\left(\frac{\pi }{4}\right)$$

where *S_i_* represents the spreading surface area (mm^2^) for the applied load *i* (g); *d_i_* represents the mean diameter (mm) of the sample under load.

#### 2.9.4. Texture Analysis

The texture analysis of the gels was investigated using a TX-700 texture analyzer (Lamy Rheology, Champagne-au-Mont-d’Or, France) fitted with a 10 kg force sensor. A two-step compression test, mimicking product application, was conducted to determine: (*i*) firmness (hardness)—maximum force required for initial deformation; (*ii*) cohesiveness—the ratio of energy between successive compressions, reflecting the structural integrity of the gel; (*iii*) springiness—The ability of the gel to regain its shape after deformation.

A sample of 30 g of each gel was placed in cylindrical containers with leveled surfaces, and the test employed a hemispherical probe. Parameters included a compression speed of 0.8 mm/s, a deformation depth of 10 mm, a trigger force of 5 g (0.05 N), and a 5 s interval between compressions. All tests were conducted at ambient temperature to simulate skin application. Triplicate analyses were performed for each formulation to ensure reproducibility.

#### 2.9.5. Antioxidant Activity

The antioxidant properties of the gels were measured using the DPPH free radical scavenging method to verify the preservation of bioactive compounds post-formulation. Approximately 1 g of each sample was mixed with 10 mL of ethanol to extract active constituents. The mixture was agitated using a vortex mixer for 5 min and filtered twice. A 0.6 mL sample was combined with 2.4 mL of DPPH solution in ethanol (0.025 g/L). Samples were incubated at room temperature, protected from light, for 30 min. The reduction in DPPH radical absorbance, indicative of antioxidant activity, was measured at 517 nm using a UV-Visible spectrophotometer (Jasco V-630, Portland, OR, USA).

#### 2.9.6. Stability

The stability of the gel formulations was monitored over a 60-day period. Samples were stored in amber glass containers at 25 °C. After this time, visual appearance, pH, and fragrance were reassessed to detect any signs of degradation or instability.

### 2.10. Preparation of Liposomes Loaded with PcF Extract

Liposomes loaded with PcF extract, coded as L_PcF, were prepared using the thin-film hydration technique followed by sonication and extrusion. The lipid phase was formulated by dissolving 100 mg of phosphatidylcholine in 10 mL of ethanol, to which 0.5 mL of PcF extract was added. The mixture was allowed to stand at 25 °C overnight to facilitate phospholipid swelling. Solvent removal was achieved using a rotary evaporator (Laboranta 4000, Heidolph Instruments GmbH & Co. KG, Kelheim, Germany) under reduced pressure at 37 °C for 2 h, resulting in the formation of a thin lipid film. This film was subsequently hydrated with distilled water at 37 °C, and the resulting dispersion was kept at 25 °C for 2 h to promote liposome stabilization. To reduce the vesicle size, the suspension underwent sonication for 20 min at 50% amplitude in an ultrasonic bath (Sonorex-Digital-10P, Bandelin-Electronic, Berlin, Germany), followed by sequential extrusion through polycarbonate membranes with pore sizes of 0.4 µm and 0.2 µm, five times each. The liposomes were separated from the unencapsulated extract by centrifugation at 10.000 rpm and 5 °C for 25 min. The pellet containing the loaded liposomes was carefully redispersed in distilled water. All samples were prepared in triplicate and stored at 4 °C until further analysis. The liposomes were characterised based on encapsulation efficiency (EE), polydispersity index (PDI), and particle size. EE was determined as the ratio of the amount of polyphenols entrapped within the liposomes to the total polyphenol content present in the initial extract. The total polyphenol content encapsulated in the liposomes was quantified using the Folin–Ciocalteu method, as previously described in the literature [21]. The PDI and average particle size were measured using a dynamic light scattering (DLS) technique with a particle size analyzer. To minimize multiple scattering effects, the liposomal dispersions were diluted with distilled water at a 1:10 ratio.

### 2.11. Formulation and Characterisation of Dermatocosmetic Products Containing Liposomes Loaded with PcF Extract

A dermatocosmetic gel containing liposomes loaded with PcF extract, coded CBG1.25-L-PcF, was prepared using 1.25% Carbopol gel matrix. The composition of the gel is presented in Table 4. The gel containing liposomes loaded with PcF extract was characterised in terms of pH, aspect, and texture, using the methods presented in Section 2.8.

### 2.12. In Vitro Polyphenols Release Study

The release behavior of polyphenols from the dermatocosmetic gel containing liposomes loaded with PcF extract (CBG1.25-L-PcF) was evaluated using a Franz diffusion cell apparatus. The donor compartment was filled with 1 g gel while the receptor compartment was filled with 50 mL of phosphate-buffered saline (PBS, 0.1 M, pH 7.4) to mimic physiological conditions. The diffusion cells were maintained at 37 °C, simulating skin temperature, with continuous stirring at 100 rpm to ensure homogeneity of the receptor phase.

At predetermined time intervals, 15, 30, 45 min, and 1, 2, 3, 4, 5, 6, 24, 48, and 72 h, 1 mL samples were withdrawn from the receptor medium and immediately replaced with an equal volume of fresh PBS to maintain sink conditions. The polyphenol concentration in each sample was quantified using UV–Vis spectrophotometry. This method enabled the assessment of the cumulative release profile of polyphenols over time, providing insights into the sustained delivery capability of the liposome-based gel compared to the formulation containing free extract (CBG1.25-PcF).

### 2.13. In Vitro Dermal Irritation and Corrosion Testing

The skin irritation and corrosion potential of a Carbopol 1.25% gel containing liposomes loaded with PcF was assessed using the OECD TG 439 (Skin Irritation) and OECD TG 431 (Skin Corrosion) guidelines. Tests were performed using EpiDerm™ reconstructed human epidermis (MatTek, Ashland, MA, USA). The gel formulation was diluted to 100 µL/mL in DMEM (Dulbecco’s Modified Eagle’s Medium) and homogenized by ultrasonication (3 × 30 min, maximum power) to ensure sterility and dispersion. DMEM was used as the negative control, and 1% SDS (sodium dodecyl sulfate) as the positive control. For the irritation and corrosion testing, the EpiDerm tissues were pre-incubated, treated with 100 µL of the test sample, and incubated for 18 ± 0.5 h at 37 °C, 5% CO_2_. After exposure, tissues were thoroughly rinsed and transferred to fresh media. Cell viability was assessed using the MTT assay, followed by isopropanol extraction and measurement of absorbance at 570 nm. Tissue viability (%) was calculated relative to the negative control.

### 2.14. Statistical Analysis

Statistical analysis was performed using the software IBM SPSS Statistics 26 (IBM Corp., Armonk, NY, USA). A *t*-test was applied to determine if there were significant differences between the samples. Pearson correlation was calculated to assess the correlation between the polyphenolic compounds and the antioxidant activity of the extracts. To highlight the relationships between phenolic compounds and phytochemical parameters, principal component analysis (PCA) was used, complementary to Pearson correlation analysis. Hierarchical cluster analysis (HCA) was applied to classify the extracts based on the similarities between their phytochemical content.

## 3. Results and Discussion

The soil in the Batoș vineyards is deep and well-structured, with a high capacity for water retention, and situated at a low altitude. The region experiences warm summers and cool nights, receiving adequate rainfall throughout the year. Additionally, a mist typically appears in late summer, which helps the grapes ripen slowly. This gradual ripening preserves the grapes’ aromas and maintains a good level of acidity. The soil of vineyards influences the chemical composition of grapes and, implicitly, along with other influencing factors (e.g., the winemaking process), the composition of the pomace.

The pH of the extract M-FN indicated its high acidity, which contributed to the pH of the mixture samples M-FN/PcF and M-FN/PcR, respectively, according to data from Table 5. The pH value influences the stability and solubility of phenolic compounds. In general, an acidic pH favors the preservation of polyphenols and prevents their oxidation, an aspect also observed in the case of the extracts analyzed in this study.

The resistivity of extracts varied within large limits. This parameter may reflect changes in the overall chemical composition, including the presence of polyphenols and their interactions with other ionic compounds.

The Total Dissolved Solids (TDS) was very high in the PcF sample (10.51 mg/L), a value that may suggest a higher concentration of soluble compounds, including polyphenols. A positive correlation trend was observed between TDS values and total polyphenols content (Table 5 and Table 6), suggesting that these contribute significantly to the chemical composition of the extracts.

In extract analysis, conductivity can be an indirect indicator of the TDS, including polyphenols and other compounds, with the highest value being determined for the PcF extract. The presence of polyphenols can indirectly influence conductivity through interactions with metal ions from extracts.

Studies show that salinity often induces an increase in polyphenol synthesis in response to oxidative stress. In extracts, the concentration of salts can affect the solubility and bioactive efficiency of polyphenols. The salinity of the hydroalcoholic extract of *P. cuspidatum* flower and root was 5.90‰ and 5.05‰, respectively, while the pomace (M-FN extract) registered the lowest value (2.10‰) for this parameter.

As expected, taking into account the role of the grape sugars in winemaking via the alcoholic fermentation, the sugar content of the pomace (M-FN) was low (1.9%). Sugars and polyphenols co-exist in plant extracts, and they can interact through non-covalent bonds, thus influencing the stability and bioavailability of the phenolic compounds.

Grape pomace extracts (Merlot and Feteasca Neagra varieties) and Japanese knotweed (*P. cuspidatum*), as well as mixtures of extracts in different proportions, were analyzed (Table 1) in terms of phytochemical screening (Total Condensed Tannins, Total Anthocyanins Content, Total Flavonoids Content, Total Polyphenols Content, Antioxidant Activity, Table 6).

### 3.1. Total Condensed Tannins

Condensed tannins are the most prevalent type of natural tannins, accounting for nearly 90% of global production [30]. These tannins are typically found in nature in a complex form with proteins, which vary depending on their chemical structure and affinity. To qualify as condensed tannins, these compounds must consist of repeating units of 3 to 8, with the precursors being a flavan-3-ol (such as catechin) or a flavan-3,4-diol (like leucoanthocyanidin). Each flavonoid comprises two phenolic rings that exhibit different reactivities and can have two configurations: with or without a hydroxyl group in symmetrical positions. These configurations lead to four distinct possibilities for the basic building blocks that form condensed tannins [31].

**Figure 1:**
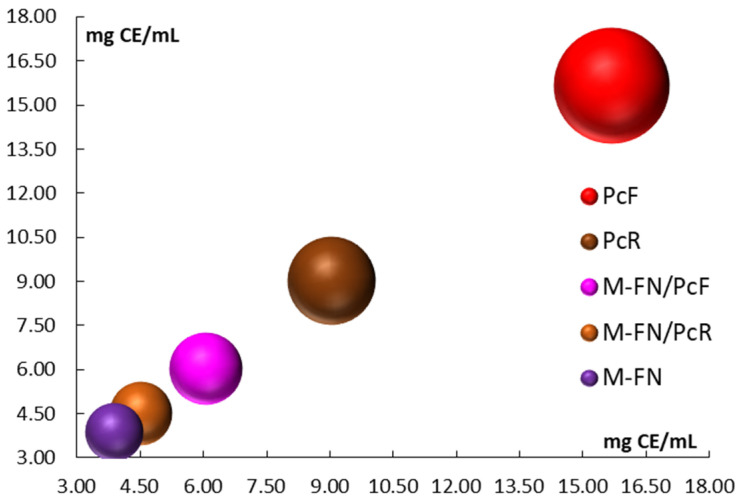
Total condensed tannins (TCT) for extracts and extract mixtures obtained from grape pomace (M-FN), roots (PcR), and flowers (PcF) of *P. cuspidatum*.

The obtained results revealed that the extracts of Japanese knotweed flower and root contained high levels of condensed tannins, with values of 15.682 ± 0.026 mg CE/mL and 9.041 ± 0.331 mg CE/mL, respectively (Table 6). These values were significantly higher compared to the other extracts analyzed (Figure 1 and Table 6). Additionally, the contribution of condensed tannins from the Japanese knotweed flower extract is evident when comparing the mixture of extracts that include this extract. For instance, the mixture M-FN/PcF showed a condensed tannin content of 6.058 ± 0.082 mg CE/mL, while M-FN alone had a lower content of 3.881 ± 0.064 mg CE/mL. Considering that the tannin content can vary depending on the plant species, the specific part of the plant, and external factors (e.g., harvesting time, season, and light intensity), certain conclusions can be drawn regarding the content of condensed tannins found in different parts of Japanese knotweed. Thus, according to studies by Furlan et al. [32], the content of tannins, which is found in most plant tissues, is in the range of 2–5% of its weight in the fresh state. However, in the dried state, the level of condensed tannins increases up to 25% of its weight, according to studies by Cuong et al. [33]. This could be one of the explanations that Japanese knotweed extracts, obtained from the dried flower and root, lead to the highest concentration of condensed tannins compared to the semi-dried grape marc extracts from which the hydroalcoholic extracts were obtained. On the other hand, Molino et al. [30] revealed that tannins are good antioxidants due to their chemical structure (i.e., benzene cycle and the phenolic hydroxyl groups), and this can stabilize free radical intermediates and maintain a balance through rapid reduction-oxidation reactions between prooxidant molecules and the tannins themselves (i.e., maintaining antioxidant/prooxidant balance) [34,35].

### 3.2. Total Anthocyanins Content

Anthocyanin pigments undergo reversible transformations as a function of pH, manifested by significant changes in the absorption spectra in the visible range. The colored oxonium form predominates at pH = 1.0 and the colorless form (hemiacetal) at pH = 4.5. The pH variation method used in this research was based on this reaction, which allowed the rapid, accurate determination of total anthocyanins even in the presence of degraded polymerized pigments or other interfering compounds [23].

**Figure 2:**
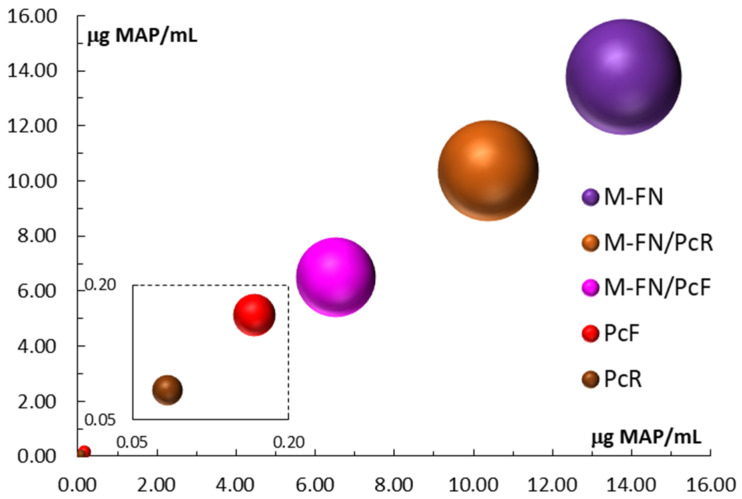
Total anthocyanin content (TAC) for extracts and extract mixtures obtained from grape pomace (M-FN), roots (PcR), and flowers (PcF) of *P. cuspidatum*.

The results from measuring the total anthocyanin content (TAC) show a high level of anthocyanins in the range of 10.659 ± 0.193 to 13.804 ± 0.293 μg MAP/mL in grape pomace extracts (Table 6 and Figure 2). A decrease in TAC is also observed in extracts made by mixing pomace extract with Japanese knotweed flower extract compared to pomace extracts alone. Very low TAC values were found for Japanese knotweed extracts, both the flower (0.167 ± 0.005 μg MAP/mL) and the root (0.083 ± 0.005 μg MAP/mL).

### 3.3. Total Flavonoids Content

**Figure 3:**
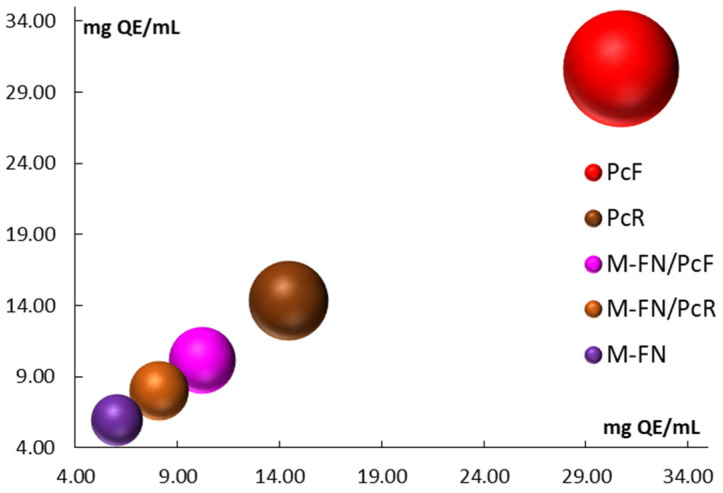
Total flavonoids content (TFC) for extracts and extract mixtures obtained from grape pomace (M-FN), roots (PcR), and flowers (PcF) of *P. cuspidatum*.

The high flavonoid content in the pomace extracts is particularly noteworthy compared to the other extracts analyzed. Among these pomace extracts, the M-FN extract stands out, with a lower TFC value of 5.976 ± 0.170 mg QE/mL (Table 6 and Figure 3). The values for the TFC of mixed extracts (i.e., grape pomace combined with *P. cuspidatum*) suggest a significant contribution from the Japanese knotweed flower extract. Specifically, the TFC values are as follows: M-FN extract (5.976 mg QE/mL), PcF extract (30.679 mg QE/mL), M-FN/PcF extract (10.178 mg QE/mL), and M-FN/PcR extract (8.056 mg QE/mL).

### 3.4. Total Polyphenols Content

**Figure 4:**
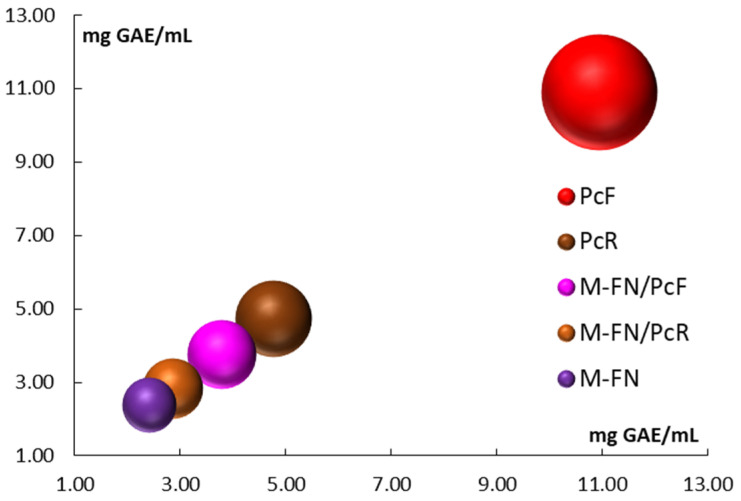
Total polyphenols content (TPC) for extracts and extract mixtures obtained from grape pomace (M-FN), roots (PcR), and flowers (PcF) of *P. cuspidatum*.

The results of the analysis clearly indicate that the Japanese knotweed flower extracts have a significantly high polyphenol content of 10,920 ± 0.268 mg GAE/mL, especially when compared to the grape pomace extract, which measures only 2399 ± 0.035 mg GAE/mL (Figure 4). Additionally, the *P. cuspidatum* root extract also shows a relatively high polyphenol content of 4751 ± 0.072 mg GAE/mL.

When examining the total phenolic content (TPC) of mixed extracts (pomace combined with Japanese knotweed), it becomes evident that the *P. cuspidatum* flower extract contributes significantly to the overall values. For instance, the mixed extract M-FN shows a TPC of 2399 ± 0.035 mg GAE/mL, while the *P. cuspidatum* flower extract (PcF) stands at 10,920 ± 0.268 mg GAE/mL, and the mixed extract of *P. cuspidatum* flower (M-FN/PcF) presents a TPC of 3769 ± 0.017 mg GAE/mL.

### 3.5. DPPH (2,2-Diphenyl-Picryl-Hydrazyl) Radical Scavenging Capacity

**Figure 5:**
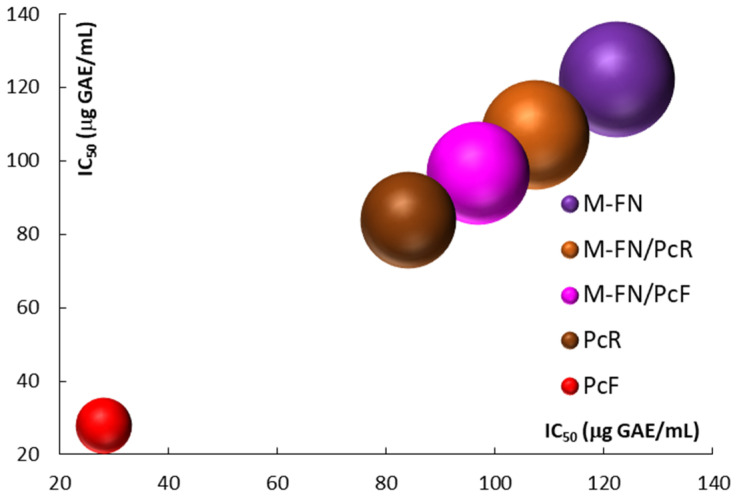
Antioxidant activity (AA) for extracts and extract mixtures obtained from grape pomace (M-FN), roots (PcR), and flowers (PcF) of *P. cuspidatum*.

The antioxidant activity, measured as IC_50_, ranges from 28.04 ± 1.12 µg GAE/mL for the extract of *P. cuspidatum* flowers to 122.29 ± 2.36 µg GAE/mL for the extract of red grape pomace (Merlot-Feteasca Neagra) (Figure 5). This variation suggests a direct correlation with the total polyphenols content (TPC), total flavonoids content (TFC), and total anthocyanins content (TAC).

### 3.6. Characterization of Extracts

#### 3.6.1. Correlation of Antioxidant Activity with Polyphenols Determined by HPLC/DAD/FLD

*T*-test was applied to determine if there are significant differences between the samples obtained from grape pomace extracts (red grape varieties) and Japanese knotweed (*P. cuspidatum*) in terms of the phenolic compounds and antioxidant activity. The total condensed tannins (TCT), the total anthocyanin content (TAC), the total flavonoid content (TFC), the total polyphenol content (TPC), as well as antioxidant activity expressed as IC_50_ differed (*p* < 0.05) among the analyzed extracts.

**Figure 6:**
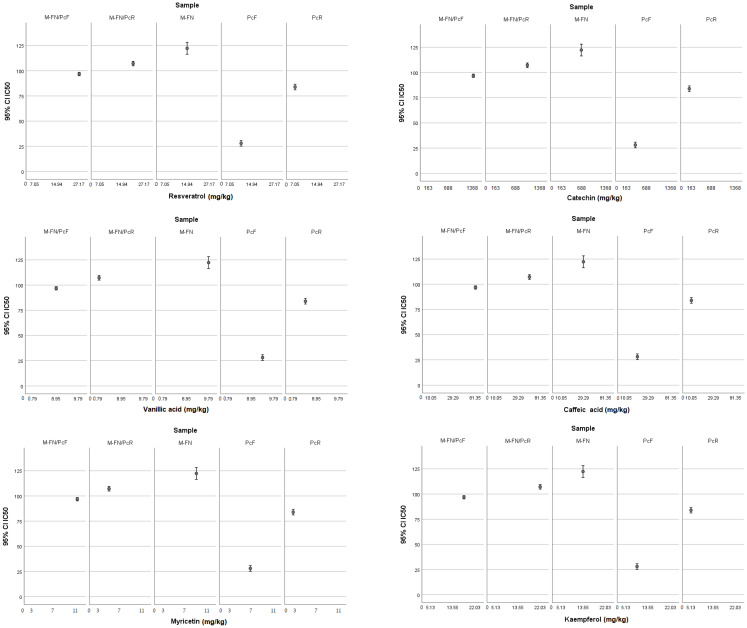
The matrices of IC_50_ extracts in relation to the six phenolic compounds investigated by HPLC.

The correlation of the antioxidant activity with each of the six polyphenols (i.e., catechin, vanillic acid, caffeic acid, myricetin, resveratrol, and kaempferol) showed a quite similar distribution of data between samples (Figure 6 and Tables S7–S11). Thus, the sample consisting of pomace of Merlot and Feteasca Neagra (M-FN) was characterized by the highest IC_50_, it being followed by the samples containing *P. cuspidatum*, namely M-FN/PcR and M-FN/PcF, respectively. The lowest IC_50_ value was determined for the extract obtained only from flowers of *P. cuspidatum* (PcF extract), whose antioxidant activity is associated with small amounts of resveratrol (9.17 mg/kg), catechin (217.62 mg/kg), caffeic acid (11.32 mg/kg), myricetin (7.04 mg/kg) and kaempferol, respectively (6.07 mg/kg) (Table S10). The highest values of resveratrol, catechin, caffeic acid, and myricetin were determined for the sample M-FN/PcF (Table S7). As expected, this extract obtained from a mixture of grape pomace and Japanese knotweed flowers exhibited an intermediate antioxidant activity within the group of samples. Some differences between the flowers and the root of *P. cuspidatum* were established in terms of polyphenol content and antioxidant activity, too. Although the smallest content of five polyphenols from the total of six determined is shown for PcR extract (resveratrol 7.05 mg/kg, catechin 162.61 mg/kg, caffeic acid 10.05 mg/kg, myricetin 3.02 mg/kg, and kaempferol 5.13 mg/kg), this sample registered a high bleaching effect, respectively, a high antioxidant activity compared with M-FN (Table S9), M-FN/PcF (Table S7), and M-FN/PcR (Table S8) extracts. According to One-Way ANOVA, all five samples differ from each other in terms of the analyzed phytochemical compounds. The distribution of data, in particular the correlation of the IC_50_ values and the concentration of the six polyphenols (i.e., catechin, vanillic acid, caffeic acid, myricetin, resveratrol, and kaempferol), emphasized that the high antioxidant values of the Japanese knotweed extracts could be due to other polyphenols that remained to be further investigated.

Peng et al. revealed that over 67 active compounds are found in the *P. cuspidatum* root extract, including quinones, stilbenes, flavonoids, coumarins, and lignans [36]. The quality of extracts from Japanese knotweed root is primarily indicated by two key compounds: emodin and polydatin. In addition, other active compounds such as resveratrol, apigenin, catechin, quercetin, and rhein (i.e., cassic acid) are well-known for improving the pharmacological activity of Japanese knotweed [37]. Dai et al. reported that the resveratrol content in the roots of *P. cuspidatum* is significantly higher than in other plants [37]. However, resveratrol primarily exists in the form of its glycoside, named polydatin. Chong et al. [38] related that the polydatin concentrations in *P. cuspidatum* are 10 to 15 times greater than those of resveratrol. In this respect, an efficient extraction and conversion of polydatin to resveratrol from *P. cuspidatum* can offer a viable method for obtaining high yields of resveratrol [39]. In recent years, *P. cuspidatum* has been found to exhibit strong estrogenic activity due to the anthraquinones it contains [16]. Additionally, other studies indicate that the stilbene content in *P. cuspidatum* is the highest among its components. While many papers have focused on analyzing the anthraquinones or stilbenes in *P. cuspidatum*, most of these studies concentrate specifically on the roots of the plant [16,36]. Consequently, there is a lack of information available regarding the chemical components of the flowers of the *P. cuspidatum* plant.

#### 3.6.2. Pearson Analysis

Table 7 reports the correlation between the polyphenolic compounds determined in all extracts and their antioxidant activity. High amounts of polyphenols and flavonoids were associated with high antioxidant capacity of the samples.

The data show the highest and inverse correlations (r > 0.95, *p* < 0.01) between IC_50_ and TCT, TFC, and TPC, respectively. As was stated previously, the Pearson analysis underlines weak and statistically significant relationships between IC_50_ and the concentration of the polyphenols determined by HPLC/DAD/FLD. Pearson’s correlation analysis of quantitative data revealed moderate correlations between IC_50_ and catechin (r = 0.569, *p* < 0.05) and between IC_50_ and kaempferol (r = 0.633, *p* < 0.05). These results support the idea that different phenolic compounds can scavenge various free radicals based on their unique chemical structures.

The TPC was positively correlated with TFC (r = 0.998, *p* < 0.01), TCT (r = 0.983, *p* < 0.01), and negatively correlated with TAC (r = 0.744, *p* < 0.01). Negative and statistically significant correlations were established between TPC and polyphenols as follows: TPC and catechin (r = −0.585, *p* < 0.05), TPC and caffeic acid (r = −0.539, *p* < 0.05), TPC and resveratrol (r = −0.534, *p* < 0.05), and TPC and kaempferol (r = −0.643, *p* < 0.01), respectively.

#### 3.6.3. Regression Analysis

**Figure 7:**
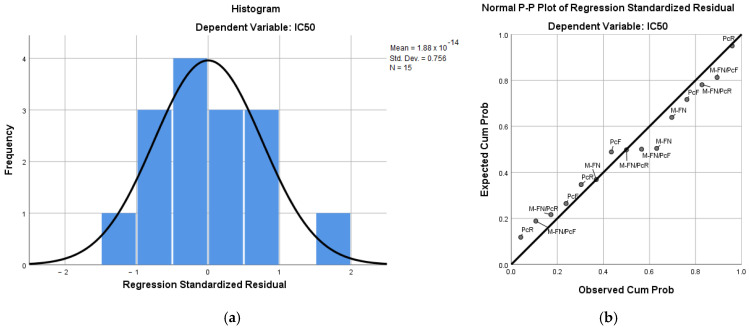
The histogram (**a**) and the Normal P-P Plot of Regression Standardized Residual (**b**).

The IC_50_ values of the five extracts were evaluated in relation to the other parameters (dependent variables), and the results were interpreted through a multiple regression model. The experimental and predicted data for IC_50_ among the extracts are shown in Figure 7. The regression model that expresses the IC_50_ (μg GAE/mL) as a function of the experimental factors is described by Equation (5).

Pred (IC50) = 222.652 − 6.274 × TCT + 7.493 × TAC + 1.255 × TPC − 5.518 × Vanillic acid + 2.215 × Caffeic acid − 14.273 × kaempferol (R squared = 0.999)(5)

Only the above six experimental factors (TCT, TAC, TPC, vanillic acid, caffeic acid, and kaempferol were included in Equation (5), while TFC, catechin, myricetin, and resveratrol were excluded from the model, probably due to their cross effects (R-squared value was very close to 1).

#### 3.6.4. Principal Component Analysis (PCA)

**Figure 8:**
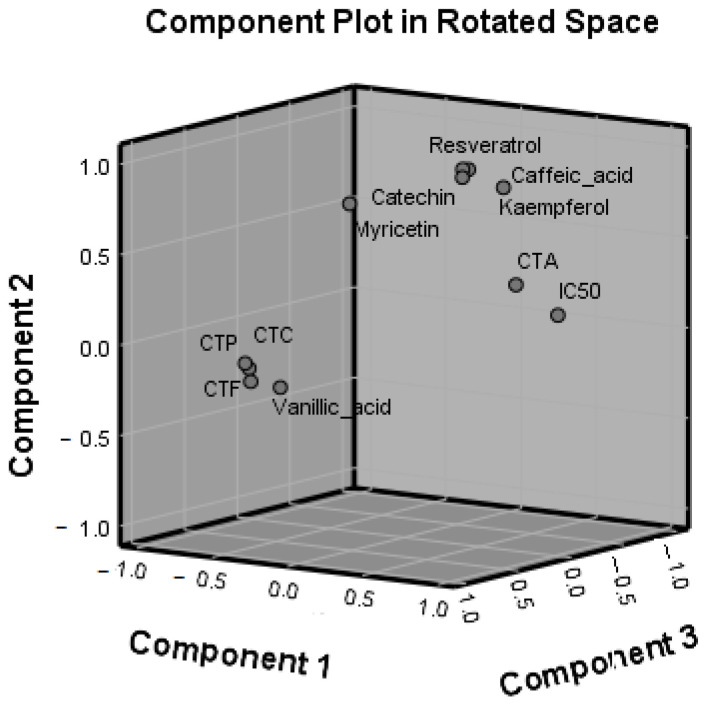
Principal Component Analysis of phenolic compounds and the antioxidant activity in the grape pomace (red grape varieties) and Japanese knotweed (*P. cuspidatum*) extracts.

To establish a descriptive model to group the five extracts based on their phenolic compounds and antioxidant activity, both PCA and HCA were performed. Principal Component Analysis summarizes the association between all dependent variables (TCT, TAC, TFC, TPC, IC_50_, and the six polyphenols). The results of Factor Analysis showed that PC1, PC2, and PC3 accounted for 96.90% of the data variance (Table 8, Figure 8).

PC1 explained a high percentage of the total variance (66.57%), TCT, TFC, and TPC being located in its negative part. Both IC_50_ and TAC, in agreement with the results of the Pearson analysis, are located in the negative part of PC1, whose structure emphasizes the strong relationships between the antioxidant activity of the five extracts and their phenolic content. As an indicator of the free radical scavenging of the extracts, the IC_50_ showed the highest factor loading. High TCT, TFC, and TPC values contribute to a high antioxidant activity of extracts, corresponding to a lower IC_50_ value.

Except for vanillic acid, the other five polyphenols determined by HPLC/DAD/FLD are located in the positive part of PC2. The interpretation of the second component loadings supports the results of the Pearson analysis. Thus, although a little expected, catechin, caffeic acid, myricetin, resveratrol, and kampherol do not covariate with IC_50_ or their correlation is not strong enough to be “clustered” together by PCA on the same component. Mapping of other polyphenols than the six determined could better clarify the relationships between their concentration and the antioxidant activity of the extracts.

The third component (PC3) discriminates the extracts only based on their vanillic acid content.

#### 3.6.5. Hierarchical Cluster Analysis

**Figure 9:**
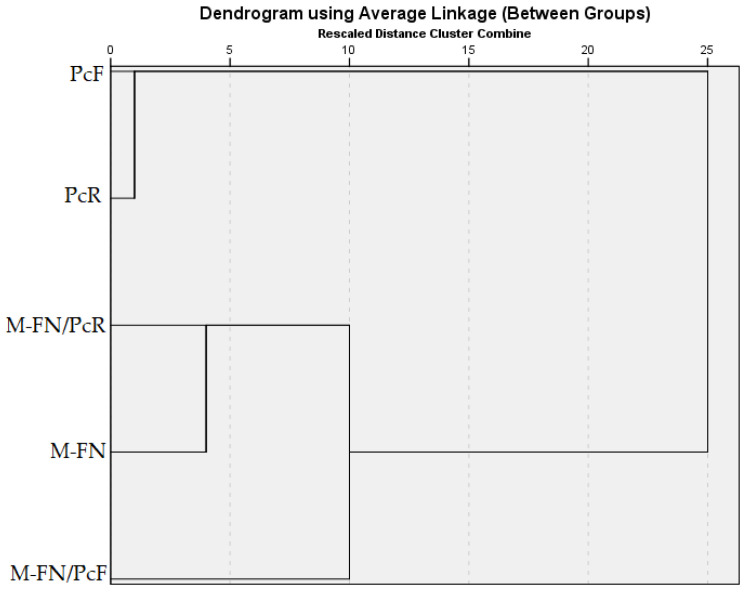
Dendrogram of variables of interest.

Hierarchical Cluster Analysis (HCA) was applied both on the values of descriptors (five extracts) and the experimental data (TCT, TFC, TPC, TAC, IC_50_, and phenolic compounds of the extracts). The Average linkage method (between groups) and the Squared Euclidean distance were used in clustering. The dendrogram, which highlights the cluster formation in hierarchical analysis, is shown in Figure 9.

The affiliation of each extract to a cluster in the first stage of clusterization, based on its phytochemical content, was obtained as follows:

Cluster 1: M-FN and M-FN/PcR (2 extracts),

Cluster 2: PcF and PcR (2 extracts).

In the second stage of hierarchical clustering analysis, the extract M-FN/PcF was added to the previously formed Cluster 1 (which included extracts M-FN and M-FN/PcR) because it showed the lowest distance coefficient to it. This suggests that M-FN/PcF is similar to M-FN and M-FN/PcR extracts in phytochemical profile, collectively forming a statistically coherent group.

The dissimilarities existing between the grape pomace extracts and the Japanese knotweed (*P. cuspidatum*) extracts are evident in Figure 9. The separation of PcF and PcR extracts may be due to their levels of TPC, TFC, and TCT, which are much higher than those in the other three extracts. The cluster formed from PcF and PcR extracts only merged in the final stage of the hierarchical analysis, indicating a significant distance between these and the cluster that resulted in the second stage (composed of extracts M-FN, M-FN/PcF, and M-FN/PcR). This suggests that they represent two well-differentiated groups, with a low degree of similarity between them, reflecting two distinct patterns in the data structure.

### 3.7. Formulation of Dermatocosmetic Products

**Figure 10:**
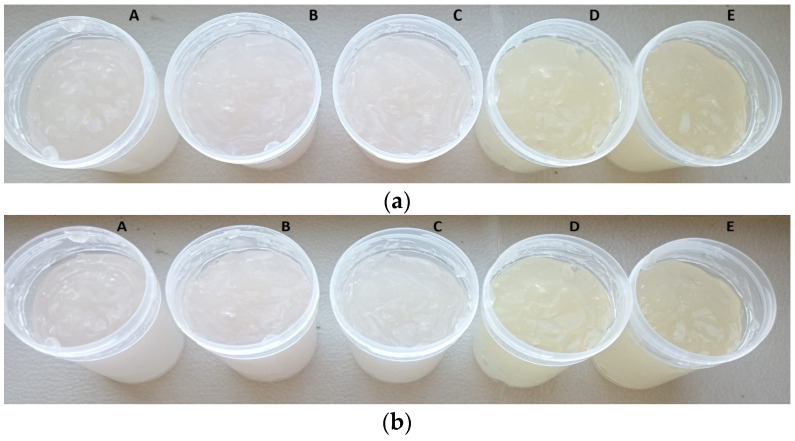
Dermatocosmetic gels formulated with 1.0% Carbopol and enriched with grape pomace and Japanese knotweed extracts at (**a**) 24 h, (**b**) 60 days; (A—CBG1-M-FN/PcF; B—CBG1-M-FN/PcR; C—CBG1-M-FN; D—CBG1-PcF; E—CBG1-PcR).

**Figure 11:**
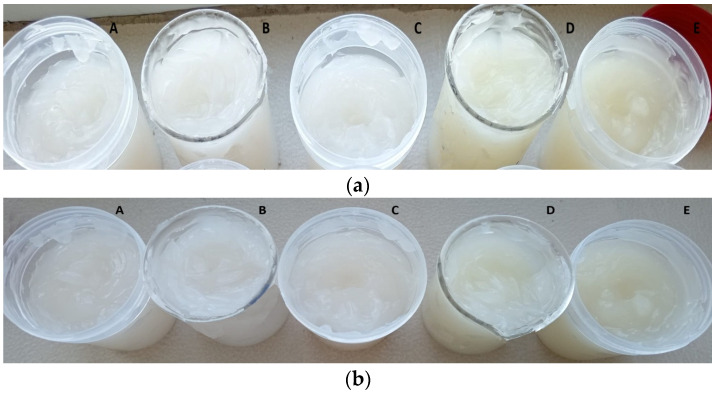
Dermatocosmetic gels formulated with 1.25% Carbopol and enriched with grape pomace and Japanese knotweed extracts at (**a**) 24 h, (**b**) 60 days; (A—CBG1.25-M-FN/PcF; B—CBG1.25-M-FN/PcR; C—CBG1.25-M-FN; D—CBG1.25-PcF; E—CBG1.25-PcR).

The stability and sensory appeal of dermatocosmetic gels are essential for consumer acceptance and long-term usability. In this study, the organoleptic properties and pH values of gels formulated with Carbopol at two concentrations (1% and 1.25%) were evaluated over a 60-day period (Figure 10 and Figure 11). Initial pH values for CBG1-based gels ranged from 5.01 to 5.22, while CBG1.25 formulations had slightly higher values between 5.20 and 5.31 after 24 h. These values fall within the optimal range for dermal applications (pH 4.5–5.5), maintaining skin compatibility and reducing the risk of irritation. Over 60 days, all formulations exhibited minimal pH changes. The slightly higher pH stability in CBG1.25 formulations may be attributed to the more robust gelling network, which could buffer interactions between Carbopol and compounds in the extracts. Across all formulations and time points, the gels demonstrated stable and homogeneous appearance, with no visible signs of phase separation, sedimentation, or texture alteration (Tables S12 and S13). This physical stability suggests effective interaction between the Carbopol polymer and the incorporated plant extracts, supporting good gel structuring and resilience to degradation over time.

The texture profile analysis (TPA) of dermatocosmetic gels formulated with Carbopol at two concentrations (1% and 1.25%) revealed differences in textural properties (Tables S14 and S19). Across all time points (24 h, 30 days, and 60 days), gels based on 1.25% Carbopol (Tables S15, S17 and S19) generally exhibited higher firmness compared to their 1% (Tables S14, S16 and S18) counterparts. Cohesiveness values remained relatively stable over the 60 days, indicating the gels’ internal bonding was largely maintained during storage, while the springiness of dermatocosmetic gels increased slightly or remained stable in time. Using the Ojeda–Arboussa method, the hydrogels demonstrated a high degree of spreadability. The results indicate that CBG 1.25% formulations had slightly higher values than CBG 1%. Therefore, the texture parameters demonstrated good temporal stability, with only minor fluctuations observed over the 60 days. Gels with 1.25% Carbopol generally showed greater firmness and elasticity retention, making them more suitable for long-term storage (Table S13).

The results demonstrate that dermatocosmetic gels formulated with 1.25% Carbopol and enriched with grape pomace and Japanese knotweed extracts maintain excellent organoleptic properties and pH stability over 60 days (Figure 11). These properties highlight the potential of these formulations for safe, effective, and consumer-acceptable cosmetic applications.

The antioxidant activity assessment revealed notable differences among the tested dermatocosmetic gel formulations, with performance strongly influenced by the type and concentration of the incorporated plant extracts (Table 9). Formulations containing PcF alone (CBG1-PcF and CBG1.25-PcF) consistently outperformed those with pomace (M-FN) or mixed extracts.

The highest antioxidant activity was observed in the CBG1.25-PcF formulation, which exhibited a % inhibition of 77.33 ± 0.52% and a Trolox equivalent (TE) value of 1.4521 ± 0.0101 mM/g. This superior activity is attributed to the high polyphenol content of the PcF extract, which had a total polyphenol content of 10.920 mg GAE/mL, significantly higher than that of the other extracts used. These results clearly highlight CBG1.25-PcF as the most effective formulation, likely due to both the higher concentration of Carbopol (1.25%), which may enhance polyphenol retention and stability, and the potent antioxidant capacity of the PcF extract. This formulation demonstrates the best potential for cosmetic applications targeting oxidative stress and skin protection.

### 3.8. Liposomes Loaded with PcF and Dermatocosmetic Gel Containing Liposomes Loaded with PcF

To improve the stability, bioavailability, and dermal delivery of the PcF, selected based on its richness in polyphenolic compounds (TPC = 10.920 mg GAE/mL), the extract was first encapsulated in liposomes prior to its incorporation into the gel formulation. Liposomal encapsulation offers a protective mechanism against environmental degradation of polyphenolic compounds, which are otherwise sensitive to oxidation and light. Moreover, liposomes facilitate enhanced skin penetration and allow for sustained release of the active compounds, thereby potentially increasing the biological efficacy of the formulation. The choice of a 1.25% Carbopol gel matrix was intentional, as it provides higher viscosity, improved texture, and better structural stability compared to lower concentrations. This concentration also ensures uniform dispersion of the liposomes and maintains a skin-compatible pH, further supporting the functional and sensorial properties of the final dermatocosmetic product.

The liposomal formulation was characterized based on EE, particle size, and PDI, as reported in Table 10. 

To assess the influence of PcF extract on liposomal characteristics, extract-loaded liposomes were compared to empty ones. The empty liposomes exhibited smaller particle sizes (100.4 ± 0.34 nm), whereas the incorporation of the extract led to a noticeable increase in particle size (157.6 ± 2.30 nm). This size enlargement is attributed to the integration of phytochemicals into the vesicle structure. The extract contains phytocompounds with varying polarities, which, as reported by Castangia et al. (2015) [40], localize differently within lipid vesicles: non-polar compounds integrate into the lipid bilayer, while polar ones are confined to the aqueous core. This spatial distribution contributes to an overall increase in vesicle size. Moreover, the insertion of non-polar compounds may induce a fluidizing effect on the membrane, disrupting phosphatidylcholine (PC) packing and promoting deeper incorporation of polyphenols into the bilayer, similar to the mechanisms observed for eugenol and isoeugenol [41]. Comparable findings have been reported in the literature, where extract incorporation led to size increases, as seen with grape seed [42] and licorice extracts [40]. The polydispersity index (PDI) reflects the uniformity of particle size distribution. PDI values below 0.1 suggest a highly homogeneous system, while values above 0.3 indicate significant heterogeneity. The PcF extract-loaded liposomes displayed a narrow size distribution and improved homogeneity, with a reduced tendency toward aggregation. In contrast, empty liposomes exhibited a broader size distribution, aligning with previous reports [20]. The EE of liposomes was found to be 84.60 ± 2.23%, consistent with values reported in similar studies. For example, Montagner et al. [43] encapsulated grape marc seed extract in liposomal vesicles, achieving a particle size of 238.6 nm, a PDI of 0.252, and an EE of 0.75%. Similarly, Popovici et al. [19] investigated the encapsulation of sea buckthorn and grape pomace extracts in liposomes, finding encapsulation efficiencies ranging from 84% to 90%, highlighting the effectiveness of liposomal systems in retaining bioactive compounds.

Table 11 presents the characteristics of the dermatocosmetic gel formulation containing liposomes loaded with *P. cuspidatum* flower extract. After 24 h, the gel exhibited a homogeneous appearance, a whitish color, and a specific aromatic smell, indicating good initial organoleptic stability. The pH was measured at 5.20 ± 0.16, within the skin-friendly range. TPA values reflected a stable and pleasant gel consistency. After 30 days of storage, the gel maintained its homogeneous appearance, color, and smell, with no observable signs of phase separation, sedimentation, or texture degradation. The pH slightly increased to 5.26 ± 0.03, remaining within acceptable limits. TPA values after 30 days revealed a minor decrease in firmness (0.311 ± 0.012 N) and springiness (0.709 ± 0.026), while cohesiveness remained virtually unchanged (0.648 ± 0.017), suggesting good long-term physical stability of the formulation.

**Figure 12:**
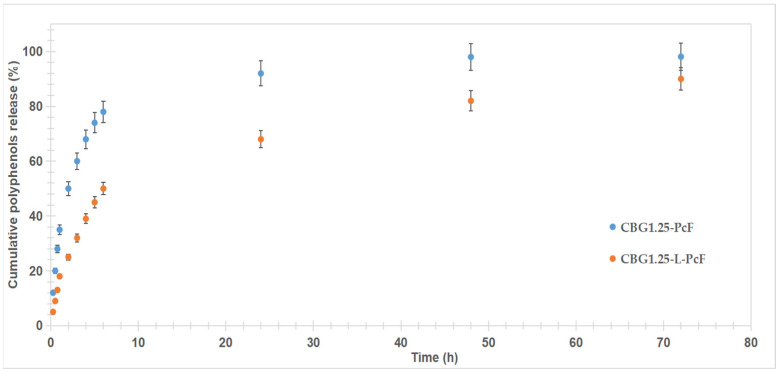
Release of polyphenols from dermatocosmetic gels.

Figure 12 presents the release behaviour of the dermatocosmetic gels. The gel with free PcF (CBG1.25-PcF) shows rapid release, reaching ~92% within the first 24 h, consistent with release behavior typical of unencapsulated extracts. In contrast, the liposome-based gel (CBG1.25-L-PcF) demonstrates controlled and sustained release, with only ~68% release at 24 h, extending up to ~90% at 72 h. This supports the role of liposomes in modulating release kinetics.

Analysis of the release kinetics of polyphenols from dermatocosmetic gels was performed by fitting several mathematical models (zero-order, first-order, Higuchi, Korsmeyer-Peppas, and Hixson-Crowell models) to determine the most accurate description of release mechanisms. The release kinetics were analyzed using KinetDS 3 software.

The mathematical models Equations (6)–(10) used are:

Zero-order model:

(6) $$M_{t}=K_{0}\cdot t$$

First-order model

(7) $$M_{t}=100\cdot \left(1-e^{-kt}\right)$$

Higuchi model:

(8) $$M_{t}=K_{H}\cdot t^{0.5}$$

Korsmeyer-Peppas model:

(9) $$M_{t}=K_{p}\cdot t^{n}$$

Hixson-Crowell model:

(10) $$M_{t}^{\frac{1}{3}}=K_{HC}\cdot t+M_{0}^{\frac{1}{3}}$$

where $M_{t}$ is the amount of polyphenols released at time *t*, $K_{0}$ is the zero-order model constant, *k* is the first-order model constant, $K_{H}$ is the Higuchi release constant, $K_{p}$ is the Korsmeyer-Peppas constant, *n* is an exponential factor, and *K_HC_* is the Hixson-Crowell release rate constant.

The best prediction model was chosen using the correlation coefficient (R^2^), root mean square error (RMSE), and Akaike criterion (AIC). A good model is considered when it has a correlation coefficient (R^2^) value close to 1, as well as lower root mean square error (RMSE) and Akaike criterion (AIC).

In Table 12 were presented R^2^, RMSE, and AIC values for the fitting models. Among the models tested, the Korsmeyer–Peppas model demonstrated a strong fit, especially for the liposome-loaded gel (R^2^ = 0.921), and the lowest RMSE and AIC values. Models like zero- and first-order kinetics, Hixson–Crowell, and Higuchi showed lower correlation and higher errors, particularly in the non-liposomal gel, indicating they may not adequately capture the complex behavior of polyphenol release from these gel matrices. The release exponent for both dermatocosmetic gels was smaller than 0.5 (*n* = 0.4862 for CBG1.25-L-PcF and *n* = 0.3486 for CBG1.25-PcF), confirming a Fickian diffusion behavior. Therefore, the inclusion of liposomes in CBG1.25-L-PcF significantly improved the release control and model fit, supporting the role of liposomes in enhancing the stability and modulation of active compound delivery.

Dermal toxicity and irritation potential were assessed using the EpiDerm™ reconstructed human epidermis model, a widely recognized in vitro alternative to animal testing. This model complies with the OECD Test Guidelines 439 (Skin Irritation) and 431 (Skin Corrosion) [44,45], and supports ethical research by adhering to the 3R principles (Replacement, Reduction, and Refinement of animal use). As the development of biocompatible and biodegradable nanocarriers gains importance in dermatocosmetic applications, evaluating the skin compatibility of liposome-based formulations is essential. To determine cytotoxic effects, EpiDerm™ tissue inserts were exposed to the test gel, followed by an MTT viability assay to assess cellular metabolic activity after treatment. The results are summarized in Table 13. No significant tissue damage or loss of viability was observed. On the contrary, a mild proliferative effect was indicated by the viability exceeding 100%. Based on the OECD TG 439 criteria, the test product did not induce any irritation on the EpiDerm tissue model. Tissue viability remained well above the 50% threshold, classifying it as Non-Irritant. According to OECD TG 431, the formulation is also non-corrosive, as the viability exceeded 15%. Moreover, the observed increase in tissue viability suggests potential biocompatibility and proliferative support, likely attributable to the encapsulated bioactive compounds in the liposomal gel.

## 4. Conclusions

The hydroalcoholic extracts of grape pomace (Merlot and Feteasca Neagra varieties) and the roots and flowers of Japanese knotweed were screened in preliminary experiments for their phenolic compounds and antioxidant activity. The dermatocosmetic gels enriched with the analyzed extracts led to safe, effective, and consumer-acceptable cosmetic formulations. Among the samples, the *P. cuspidatum* flower extract demonstrated good antioxidant activity and showed the greatest potential as a source of compounds for dermatocosmetic applications. Its encapsulation in liposomes prior to incorporation into the gel formulation improved the stability, bioavailability, and dermal delivery of the bioactive compounds. A non-irritant, non-corrosive, and good long-term physical stability formulation was this way designed in our research.

This study demonstrated that the analyzed plant extracts could be viable ingredients for cosmetic formulations. Moreover, the use of grape pomace fits into the growing context of the circular economy, while *P. cuspidatum* is a medicinal plant exhibiting an extremely valuable phytochemical profile. These results encourage their use as sustainable sources of functional compounds in the dermatocosmetic industry.

## 5. Patents

Radulescu C., Olteanu R.L., Pavaloiu R.D., Sha’at F., Petrescu M.M., Menihart L., Moldovan D., Gel dermatocosmetic pe baza de lipozomi cu extract de floare de *Polygonum cuspidatum* (Dermatocosmetic gel based on liposomes with *Polygonum cuspidatum* flower extract) patent application A/00383/2025 (national patent OSIM).

## Figures and Tables

**Table 1:** Assigned the code of the hydroalcoholic extract samples.

Sample Code	Extract
M-FN/PcF	Mixture of Merlot—Feteasca Neagra grapes pomace and *P. cuspidatum* flower hydroalcoholic extract, in a ratio of 1:1 (*v*:*v*)
M-FN/PcR	Mixture of Merlot—Feteasca Neagra grape pomace extract and *P. cuspidatum* root hydroalcoholic extract, in a 3:1 ratio (*v*:*v*)
M-FN	Merlot—Feteasca Neagra grape pomace hydroalcoholic extract
PcF	Hydroalcoholic extract of *P. cuspidatum* flower
PcR	Hydroalcoholic extract of *P. cuspidatum* root

**Table 2:** Composition of the first gel-based cosmetic formulations enriched with grape pomace (Feteasca Neagra and Merlot varieties) and *P. cuspidatum* extracts.

Component	Formulations				
	CBG1-M-FN/PcF	CBG1-M-FN/PcR	CBG1-M-FN	CBG1-PcF	CBG1-PcR
Carbopol 940	1%	1%	1%	1%	1%
Glycerin	6%	6%	6%	6%	6%
M-FN/PcF	2%	-	-	-	-
M-FN/PcR	-	2%	-	-	-
M-FN	-	-	2%	-	-
PcF	-	-	-	2%	-
PcR	-	-	-	-	2%
Lavandin essential oil	1%	1%	1%	1%	1%
Sodium hydroxide	q.s	q.s	q.s	q.s	q.s
Purified water	Up to 100%	Up to 100%	Up to 100%	Up to 100%	Up to 100%

**Table 3:** Composition of second gel-based cosmetic formulations enriched with grape pomace (Feteasca Neagra and Merlot varieties) and *P. cuspidatum* extracts.

Component	Formulations				
	CBG1.25-M-FN/PcF	CBG1.25-M-FN/PcR	CBG1.25-M-FN	CBG1.25-PcF	CBG1.25-PcR
Carbopol 940	1.25%	1.25%	1.25%	1.25%	1.25%
Glycerin	7.5%	7.5%	7.5%	7.5%	7.5%
M-FN/PcF	2%	-	-	-	-
M-FN/PcR	-	2%	-	-	-
M-FN	-	-	2%	-	-
PcF	-	-	-	2%	-
PcR	-	-	-	-	2%
Lavandin essential oil	1%	1%	1%	1%	1%
Sodium hydroxide	q.s.	q.s.	q.s.	q.s.	q.s.
Purified water	Up to 100%	Up to 100%	Up to 100%	Up to 100%	Up to 100%

**Table 4:** Composition of the dermatocosmetic gel containing liposomes loaded with PcF extract (CBG1.25-L-PcF).

Component	Component
Carbopol 940	1.25%
Glycerin	7.5%
Liposomes containing PcF	2%
Vitamin E	0.5%
Lavandin essential oil	1%
Sodium hydroxide	q.s.
Purified water	Up to 100%

**Table 5:** Electrochemical characterization of hydroalcoholic extracts.

Sample	pH	Rezistivity [Ω/cm]	TDS [mg/L]	Conductivity [µS/cm]	Salinity [‰]	Sugar Content [%]
M-FN/PcF	4.118	138.7	7.21	7.22	3.90	2.6
M-FN/PcR	3.740	181.7	5.51	5.52	3.00	2.4
M-FN	3.863	250	4.01	4.01	2.10	1.9
PcF	4.194	95.3	10.51	10.52	5.90	3.1
PcR	3.620	79.8	8.02	8.03	5.05	2.9

**Table 6:** Phytochemical screening data of hydroalcoholic extracts.

Sample Code	Total Condensed Tannins (TCT) [mg CE/mL]	Total Anthocyanins Content (TAC) MAP * [μg/mL]	Total Flavonoids Content (TFC) [mg QE/mL]	Total Polyphenols Content (TPC) [mg GAE/mL]	DPPH Radical Scavenging Capacity, IC_50_ [μg GAE/mL]
M-FN/PcF	6.058 ± 0.082	6.513 ± 0.334	10.178 ± 0.098	3.769 ± 0.017	96.77 ± 0.67
M-FN/PcR	4.523 ± 0.106	10.381 ± 0.048	8.056 ± 0.226	2.843 ± 0.037	107.17 ± 0.92
M-FN	3.881 ± 0.064	13.804 ± 0.293	5.976 ± 0.170	2.399 ± 0.035	122.29 ± 2.36
PcF	15.682 ± 0.026	0.167 ± 0.005	30.679 ± 0.393	10.920 ± 0.268	28.04 ± 1.12
PcR	9.041 ± 0.331	0.083 ± 0.005	14.396 ± 0.129	4.751 ± 0.072	83.91 ± 1.13

* Concentration of monomeric anthocyanin pigment (MAP). Note: Results are expressed as mean ± standard deviation (triplicate).

**Table 7:** Pearson correlation analysis between the antioxidant activity (expressed as IC_50_) and the polyphenolic compounds among the extracts obtained from grape pomace and Japanese knotweed.

TCT		TAC	TFC	TPC	IC_50_	Catechin	Vanillic_Acid	Caffeic_Acid	Myricetin	Resveratrol	Kaempferol
**TCT**	1	−0.846 **	0.992 **	0.983 **	−0.988 **	−0.668 **	0.299	−0.611 *	−0.235	−0.623 *	−0.737 **
**TAC**		1	−0.785 **	−0.748 **	0.827 **	0.603 *	−0.159	0.503	0.414	0.570 *	0.724 **
**TFC**			1	0.998 **	−0.993 **	−0.614 *	0.275	−0.559 *	−0.167	−0.562 *	−0.667 **
**TPC**				1	−0.987 **	−0.585 *	0.305	−0.539 *	−0.111	−0.534 *	−0.643 **
**IC50**					1	0.569 *	−0.232	0.499	0.161	0.512	0.633 *
**Catechin**						1	−0.189	0.978 **	0.755 **	0.991 **	0.925 **
**Vanillic_acid**							1	−0.353	0.408	−0.287	−0.488
**Caffeic_acid**								1	0.650 **	0.993 **	0.937 **
**Myricetin**									1	0.718 **	0.545 *
**Resveratrol**										1	0.947 **
**Kaempferol**											1

** Correlation is significant at the 0.01 level (2-tailed). * Correlation is significant at the 0.05 level (2-tailed).

**Table 8:** Factor loadings (Varimax normalized) using principal component extraction.

	Component		
	PC1	PC2	PC3
**Eigenvalue**	7.323	2.079	1.257
**Cumulative variance (%)**	66.571	85.472	96.90
**TCT**	−0.937	−0.327	0.121
**TAC**	0.796	0.377	0.076
**TFC**	−0.951	−0.257	0.115
**TPC**	−0.948	−0.222	0.158
**IC_50_**	0.975	0.207	−0.066
**Catechin**	0.384	0.917	−0.002
**Vanillic_acid**	−0.146	−0.157	0.969
**Caffeic_acid**	0.296	0.926	−0.188
**Myricetin**	0.034	0.819	0.568
**Resveratrol**	0.316	0.941	−0.104
**Kaempferol**	0.462	0.829	−0.281

Extraction Method: Principal Component Analysis. Rotation Method: Varimax with Kaiser Normalization ^a^. a. Rotation converged in 4 iterations.

**Table 9:** Antioxidant activity of dermatocosmetic gels.

Sample Code	%Inhibition (Mean ± SD)	Trolox Eq. (mM/g) ± SD
CBG1-M-FN/PcF	22.75 ± 0.15%	0.4079 ± 0.0029
CBG1.25-M-FN/PcF	28.27 ± 0.40%	0.5136 ± 0.0077
CBG1-M-FN/PcR	12.62 ± 0.03%	0.2114 ± 0.0006
CBG1.25-M-FN/PcR	13.51 ± 0.04%	0.2286 ± 0.0008
CBG1-M-FN	10.54 ± 0.04%	0.1715 ± 0.0008
CBG1.25-M-FN	9.85 ± 0.02%	0.1584 ± 0.0004
CBG1-PcF	**75.71 ± 0.17%**	**1.4312 ± 0.0033**
CBG1.25-PcF	**77.33 ± 0.52%**	**1.4521 ± 0.0101**
CBG1-PcR	34.49 ± 0.17%	0.6333 ± 0.0033
CBG1.25-PcR	36.50 ± 0.27%	0.6728 ± 0.0052

**Table 10:** Characteristics of liposomes loaded with PcF extract.

Sample	Particle Size [nm]	PDI	EE [%]
L_PcF	157.6 ± 2.30	0.186 ± 0.01	84.60 ± 2.23
Empty liposome	100.4 ± 0.34	0.300 ± 0.03	-

**Table 11:** Characteristics of dermatocosmetic gel containing liposomes loaded with PcF extract.

Characteristics	CBG1.25-L-PcF
Organoleptic evaluation—after 24 h	Appearance: Homogeneous Color: whitish Smell: Aromatic, specific
pH—after 24 h	5.20 ± 0.16
TPA profile—after 24 h	Firmness (hardness): 0.327 ± 0.014 N Cohesiveness: 0.645 ± 0.011 Springiness: 0.794 ± 0.024
Organoleptic evaluation—after 30 days	Appearance: Homogeneous Color: whitish Smell: Aromatic, specific No signs of phase separation, sedimentation, or texture alteration
pH—after 30 days	5.26 ± 0.03
TPA profile—after 30 days	Firmness (hardness): 0.311 ± 0.012 N Cohesiveness: 0.648 ± 0.017 Springiness: 0.709 ± 0.026

**Table 12:** R^2^, RSME, and AIC of the fitted experimental data.

Kinetic Model	Model Coefficients	Formulation	
		CBG1.25-PcF	CBG1.25-L-PcF
*Zero-order*	R^2^	0.5171	0.7672
	RMSE	20.2123	13.1099
	AIC	105.97	95.58
*First-order*	R^2^	0.337	0.4598
	RMSE	24.2779	20.8165
	AIC	110.37	106.68
*Higuchi*	R^2^	0.7174	0.7113
	RMSE	45.4721	14.6013
	AIC	125.43	98.17
*Korsmeyer-Peppas*	R^2^	0.8382	0.9205
	RMSE	17.831	12.9263
	AIC	102.96	95.24
*Hixson-Cromwell*	R^2^	0.3978	0.5729
	RMSE	22.1349	16.4867
	AIC	108.15	101.08

**Table 13:** Evaluation of dermal irritation and corrosivity of the dermatocosmetic gel containing liposomes using the EpiDerm™ model.

Sample	Mean OD	SD	Viability [%]	Observation *^,^**
DMEM (Control)	1.261	0.049	100	-
Gel with Liposomal PcF (CBG1.25-L-PcF)	1.371	0.027	108.72	Non-Irritant (NI), Non-corrosive

* Skin Irritation prediction: tissue viability ≤50% = Irritant; >50% = Non-Irritant. ** Skin Corrosion prediction: viability ≤15% = Corrosive; >15% = Non-Corrosive.

## Data Availability

The original contributions presented in the study are included in the article and Supplementary Materials, further inquiries can be directed to the corresponding author.

## Supplementary Materials

The following supporting information can be downloaded at: https://www.mdpi.com/article/10.3390/antiox14101182/s1, Table S1. Standard polyphenols used for stock solutions for calibration; Table S2. Calibration data of standard polyphenols (0.2 ppm)—HPLC/DAD/FLD; Table S3. Calibration data of standard polyphenols (1.0 ppm)—HPLC/DAD/FLD; Table S4. Calibration data of standard polyphenols (2.5 ppm)—HPLC/DAD/FLD; Table S5. Calibration data of standard polyphenols (5 ppm)—HPLC/DAD/FLD; Table S6. Calibration data of standard polyphenols (10 ppm)—HPLC/DAD/FLD; Table S7. Chromatographic data of polyphenols in the M-FN/PcF extract; Table S8. Chromatographic data of the six polyphenols in the M-FN/PcR extract; Table S9. Chromatographic data of the six polyphenols in the M-FN extract; Table S10. Chromatographic data of the six polyphenols in the PcF extract; Table S11. Chromatographic data of the six polyphenols in the PcR extract; Table S12. Characteristics of the first gel-based cosmetic formulation enriched with grape pomace (Feteasca Neagra and Merlot varieties) and Japanese knotweed (*Polygonum cuspidatum*) extracts; Table S13. Characteristics of the second gel-based cosmetic formulation enriched with grape pomace (Feteasca Neagra and Merlot varieties) and Japanese knotweed (*Polygonum cuspidatum*) extracts; Table S14. TPA profile of the first gel-based cosmetic formulation enriched with grape pomace (Feteasca Neagra and Merlot varieties) and Japanese knotweed (*Polygonum cuspidatum*) extracts (24 h); Table S15. TPA profile of the second gel-based cosmetic formulation enriched with grape pomace (Feteasca Neagra and Merlot varieties) and Japanese knotweed (*Polygonum cuspidatum*) extracts (24 h); Table S16. TPA profile of the first gel-based cosmetic formulation enriched with grape pomace (Feteasca Neagra and Merlot varieties) and Japanese knotweed (*Polygonum cuspidatum*) extracts (30 days); Table S17. TPA profile of the second gel-based cosmetic formulation enriched with grape pomace (Feteasca Neagra and Merlot varieties) and Japanese knotweed (*Polygonum cuspidatum*) extracts (30 days); Table S18. TPA profile of the first gel-based cosmetic formulation enriched with grape pomace (Feteasca Neagra and Merlot varieties) and Japanese knotweed (*Polygonum cuspidatum*) extracts (60 days); Table S19. TPA profile of the second gel-based cosmetic formulation enriched with grape pomace (Feteasca Neagra and Merlot varieties) and Japanese knotweed (*Polygonum cuspidatum*) extracts (60 days).
