# Supplementary material for: Development and Characterization of Liposome-Based Dermocosmetic Formulations with Red Grape Pomace and Polygonum cuspidatum Extracts
Source: Antioxidants (Basel). 2025 Sep 28;14(10):1182. doi: 10.3390/antiox14101182 (PMC12561438; doi:10.3390/antiox14101182)
Supplement: Supplementary file 1 [file antioxidants-14-01182-s001.zip › antioxidants-3883172-supplementary-new.pdf]

antioxidants

Supplementary Material contents:

 https://doi.org/10.3390/antiox14101182

Table S1. Standard polyphenols used for stock solutions for calibration.

No	Polyphenols	Weight [mg]	Concentration [ppm]	Volume [µL]
1.	(+)Catechin	12.5	1250	100
2.	Caffeic acid	21.6	2160	50
3.	Vanillic acid	22.4	2240	50
4.	Kaempferol	5.0	500	200
5.	Myricetin	4.1	410	200
6.	Resveratrol	13.2	1320	100

Table S2. Calibration data of standard polyphenols (0.2 ppm) – HPLC/DAD/FLD.

Name	RT [min]	Area	RF	Calibration Amount [ppm]	Amount [ppm]	Concentration [mg/kg]
(+)Catechin	12.216	0.677	2.286	0.250	0.296	0.2959
Vanillic acid	13.168	1.954	7.562	0.224	0.258	0.2584
Caffeic acid	13.390	7.511	31.659	0.216	0.237	0.2373
Myricetin	19.663	3.123	18.163	0.164	0.172	0.1719
Resveratrol	20.360	11.106	39.020	0.262	0.285	0.2846
Kaempferol	22.250	5.114	23.058	0.200	0.222	0.2218

Table S3. Calibration data of standard polyphenols (1.0 ppm) – HPLC/DAD/FLD.

Name	RT [min]	Area	RF	Calibration Amount [ppm]	Amount [ppm]	Concentration [mg/kg]
(+)Catechin	12.207	2.744	2.376	1.250	1.155	1.1546
Vanillic acid	13.156	8.443	7.925	1.120	1.065	1.0654
Caffeic acid	13.377	32.336	30.318	1.080	1.067	1.0665
Myricetin	19.656	14.408	17.939	0.820	0.803	0.8032
Resveratrol	20.355	47.462	36.931	1.310	1.285	1.2852
Kaempferol	22.247	21.366	21.820	1.000	0.979	0.9792

Table S4. Calibration data of standard polyphenols (2.5 ppm) – HPLC/DAD/FLD.

Name	RT [min]	Area	RF	Calibration Amount [ppm]	Amount [ppm]	Concentration [mg/kg]
(+)Catechin	12.205	7.511	2.396	3.125	3.135	3.1347
Vanillic acid	13.152	22.571	7.997	2.800	2.822	2.8224
Caffeic acid	13.373	80.756	30.087	2.700	2.684	2.6841
Myricetin	19.656	36.526	17.902	2.050	2.040	2.0404
Resveratrol	20.355	119.150	36.571	3.275	3.258	3.2581
Kaempferol	22.245	53.856	21.600	2.500	2.493	2.4934

Table S5. Calibration data of standard polyphenols (5 ppm) – HPLC/DAD/FLD.

Name	RT [min]	Area	RF	Calibration Amount [ppm]	Amount [ppm]	Concentration [mg/kg]
(+)Catechin	12.196	15.101	2.402	6.250	6.287	6.2873
Vanillic acid	13.142	44.637	8.019	5.600	5.566	5.5664
Caffeic acid	13.362	161.601	30.011	5.400	5.385	5.3848
Myricetin	19.655	73.397	17.889	4.100	4.103	4.1028
Resveratrol	20.355	238.293	36.453	6.550	6.537	6.5370
Kaempferol	22.245	107.600	21.528	5.000	4.998	4.9981

Table S6. Calibration data of standard polyphenols (10 ppm) – HPLC/DAD/FLD.

Name	RT [min]	Area	RF	Calibration Amount [ppm]	Amount [ppm]	Concentration [mg/kg]
(+)Catechin	12.194	30.029	2.405	12.500	12.488	12.4876
Vanillic acid	13.140	90.065	8.030	11.200	11.216	11.2160
Caffeic acid	13.359	324.071	29.973	10.800	10.812	10.8123
Myricetin	19.653	146.685	17.883	8.200	8.202	8.2023
Resveratrol	20.351	477.217	36.394	13.100	13.112	13.1124
Kaempferol	22.244	215.014	21.493	10.000	10.004	10.0040

Table S7. Chromatographic data of polyphenols in the M-FN/PcF extract.

No	Polyphenol	Detector	RT [min]	Area	Area 100%	Height	Height 100%	Amount [ppm]	Concentration [mg/kg]	S/N	Symmetry	Tailing	Plates EP	Width 50%[min]
1.	(+)Catechin	DAD	12.157	329.23	100.000	110.8	100.00	136.762	1367.623	1116	0.92	0.97	342489	0.049
2.	Vanillic acid	FLD	13.097	7.07	100.000	1.2	100.00	0.894	8.945	120	0.79	1.16	119868	0.089
3.	Caffeic acid	DAD	13.246	124.20	100.000	25.8	100.00	8.135	81.353	107	0.94	1.27	182091	0.073
4.	Myricetin	DAD	19.632	19.79	87.753	6.1	89.04	1.104	11.042	17	0.88	1.12	888220	0.049
5.	Resveratrol	DAD	20.331	63.15	100.000	17.8	100.00	2.717	27.168	72	0.84	0.87	810529	0.053
6.	Kaempferol	DAD	22.228	19.83	81.814	5.9	82.25	1.907	19.075	16	0.95	1.09	1046818	0.051

Table S8. Chromatographic data of the six polyphenols in the M-FN/PcR extract.

No	Polyphenol	Detector	RT [min]	Area	Area 100%	Height	Height 100%	Amount [ppm]	Concentration [mg/kg]	S/N	Symmetry	Tailing	Plates EP	Width 50%[min]
1.	(+)Catechin	DAD	12.144	241.99	100.000	70.8	100.00	100.528	1005.285	375	0.97	0.95	286691	0.053
2.	Vanillic acid	FLD	13.099	0.51	100.000	0.1	100.00	0.079	0.792	11	0.86	0.88	157033	0.078
3.	Caffeic acid	DAD	13.227	203.50	100.000	42.4	100.00	6.784	67.844	260	0.99	0.97	185082	0.072
4.	Myricetin	DAD	19.619	0.25	100.000	0.1	100.00	0.611	6.110	0	0.91	1.02	1035768	0.045
5.	Resveratrol	DAD	20.324	12.27	100.000	3.5	100.00	2.317	23.166	20	1.00	0.81	800489	0.053
6.	Kaempferol	DAD	22.222	47.61	89.835	14.3	90.20	2.202	22.025	59	0.93	1.08	1058479	0.051

Table S9. Chromatographic data of the six polyphenols in the M-FN extract.

No	Polyphenol	Detector	RT [min]	Area	Area 100%	Height	Height 100%	Amount [ppm]	Concentration [mg/kg]	S/N	Symmetry	Tailing	Plates EP	Width 50%[min]
1.	(+)Catechin	DAD	12.171	69.27	100.000	24.1	100.000	68.968	687.876	94	0.86	0.99	364369	0.047
2.	Vanillic acid	FLD	13.118	6.14	100.000	1.0	100.000	0.979	9.790	73	0.84	1.15	114955	0.091
3.	Caffeic acid	DAD	13.267	64.15	100.000	14.6	100.000	2.929	29.292	40	0.86	1.08	205464	0.069
4.	Myricetin	DAD	19.642	14.42	100.000	4.4	100.000	0.804	8.036	9	0.86	1.16	866622	0.050
5.	Resveratrol	DAD	20.341	44.13	100.000	12.4	100.000	1.493	14.935	33	0.86	0.87	825717	0.053
6.	Kaempferol	DAD	22.236	7.96	62.784	2.3	62.77	1.354	13.545	5	0.95	1.01	1017953	0.052

Table S10. Chromatographic data of the six polyphenols in the PcF extract.

No	Polyphenol	Detector	RT [min]	Area	Area 100%	Height	Height 100%	Amount [ppm]	Concentration [mg/kg]	S/N	Symmetry	Tailing	Plates EP	Width 50%[min]
1.	(+)Catechin	DAD	12.157	329.23	100.000	110.8	100.00	21.762	217.623	1116	0.92	0.97	342489	0.049
2.	Vanillic acid	FLD	13.097	7.07	100.000	1.2	100.00	0.898	8.980	120	0.79	1.16	119868	0.089
3.	Caffeic acid	DAD	13.246	124.20	100.000	25.8	100.00	1.131	11.315	107	0.94	1.27	182091	0.073
4.	Myricetin	DAD	19.632	19.79	87.753	6.1	89.04	0.704	7.042	17	0.88	1.12	888220	0.049
5.	Resveratrol	DAD	20.331	63.15	100.000	17.8	100.00	0.917	9.170	72	0.84	0.87	810529	0.053
6.	Kaempferol	DAD	22.228	19.83	81.814	5.9	82.25	0.607	6.070	16	0.95	1.09	1046818	0.051

Table S11. Chromatographic data of the six polyphenols in the PcR extract.

No	Polyphenol	Detector	RT [min]	Area	Area 100%	Height	Height 100%	Amount [ppm]	Concentration [mg/kg]	S/N	Symmetry	Tailing	Plates EP	Width 50%[min]
1.	(+)Catechin	DAD	12.157	329.23	100.000	110.8	100.00	16.261	162.610	1116	0.92	0.97	342489	0.049
2.	Vanillic acid	FLD	13.097	7.07	100.000	1.2	100.00	0.671	6.714	120	0.79	1.16	119868	0.089
3.	Caffeic acid	DAD	13.246	124.20	100.000	25.8	100.00	1.005	10.052	107	0.94	1.27	182091	0.073
4.	Myricetin	DAD	19.632	19.79	87.753	6.1	89.04	0.302	3.022	17	0.88	1.12	888220	0.049
5.	Resveratrol	DAD	20.331	63.15	100.000	17.8	100.00	0.704	7.046	72	0.84	0.87	810529	0.053
6.	Kaempferol	DAD	22.228	19.83	81.814	5.9	82.25	0.513	5.130	16	0.95	1.09	1046818	0.051

Table S12. Characteristics of the first gel-based cosmetic formulation enriched with grape pomace (Feteasca Neagra and Merlot varieties) and Japanese knotweed (*Polygonum cuspidatum*) extracts.

**Characteristics**	**CBG1-M-FN/PcF**	**CBG1-M-FN/PcR**	**CBG1-M-FN**	**CBG1-PcF**	**CBG1-PcR**
Organoleptic evaluation - after 24 h	Appearance: Homogeneous<br>Color: yellowish<br>Smell: Aromatic, specific	Appearance: Homogeneous<br>Color: light yellow<br>Smell: Aromatic, specific	Appearance: Homogeneous<br>Color: light yellow<br>Smell: Aromatic, specific	Appearance: Homogeneous<br>Color: burnt yellow<br>Smell: Aromatic, specific	Appearance: Homogeneous<br>Color: mustard yellow<br>Smell: Aromatic, specific
pH - after 24 h	5.05 ± 0.03	5.22 ± 0.05	5.01 ± 0.03	5.14 ± 0.03	5.12 ± 0.03
Organoleptic evaluation - after 30 days, room temperature	Appearance: Homogeneous<br>Color: yellowish<br>Smell: Aromatic, specific<br>No signs of phase separation, sedimentation, or texture alteration	Appearance: Homogeneous<br>Color: light yellow<br>Smell: Aromatic, specific<br>No signs of phase separation, sedimentation, or texture alteration,	Appearance: Homogeneous<br>Color: light yellow<br>Smell: Aromatic, specific<br>No signs of phase separation, sedimentation, or texture alteration	Appearance: Homogeneous<br>Color: burnt yellow<br>Smell: Aromatic, specific<br>No signs of phase separation, sedimentation, or texture alteration	Appearance: Homogeneous<br>Color: mustard yellow<br>Smell: Aromatic, specific<br>No signs of phase separation, sedimentation, or texture alteration
pH - after 30 days, room temperature	5.10 ± 0.04	5.31 ± 0.03	5.08 ± 0.04	5.20 ± 0.04	5.17 ± 0.04
Organoleptic evaluation - after 60 days, room temperature	Appearance: Homogeneous<br>Color: yellowish<br>Smell: Aromatic, specific<br>No signs of phase separation, sedimentation, or texture alteration	Appearance: Homogeneous<br>Color: light yellow<br>Smell: Aromatic, specific<br>No signs of phase separation, sedimentation, or texture alteration,	Appearance: Homogeneous<br>Color: light yellow<br>Smell: Aromatic, specific<br>No signs of phase separation, sedimentation, or texture alteration	Appearance: Homogeneous<br>Color: burnt yellow<br>Smell: Aromatic, specific<br>No signs of phase separation, sedimentation, or texture alteration	Appearance: Homogeneous<br>Color: mustard yellow<br>Smell: Aromatic, specific<br>No signs of phase separation, sedimentation, or texture alteration
pH - after 60 days, room temperature	5.10 ± 0.14	5.32 ± 0.09	5.09 ± 0.06	5.20 ± 0.09	5.17 ± 0.05

Table S13. Characteristics of the second gel-based cosmetic formulation enriched with grape pomace (Feteasca Neagra and Merlot varieties) and Japanese knotweed (*Polygonum cuspidatum*) extracts.

**Characteristics**	**CBG1.25-M-FN/PcF**	**CBG1.25-M-FN/PcR**	**CBG1.25-M-FN**	**CBG1.25-PcF**	**CBG1.25-PcR**
Organoleptic evaluation - after 24 h	Appearance: Homogeneous<br>Color: pale yellow<br>Smell: Aromatic, specific	Appearance: Homogeneous<br>Color: pale yellow<br>Smell: Aromatic, specific	Appearance: Homogeneous<br>Color: light cream<br>Smell: Aromatic, specific	Appearance: Homogeneous<br>Color: amber yellow<br>Smell: Aromatic, specific	Appearance: Homogeneous<br>Color: pale yellow<br>Smell: Aromatic, specific
pH - after 24 h	5.25 ± 0.02	5.31 ± 0.03	5.20 ± 0.02	5.26 ± 0.04	5.28 ± 0.03
Organoleptic evaluation - after 30 days, room temperature	Appearance: Homogeneous<br>Color: pale yellow<br>Smell: Aromatic, specific<br>No signs of phase separation, sedimentation, or texture alteration	Appearance: Homogeneous<br>Color: pale yellow<br>Smell: Aromatic, specific<br>No signs of phase separation, sedimentation, or texture alteration,	Appearance: Homogeneous<br>Color: light cream<br>Smell: Aromatic, specific<br>No signs of phase separation, sedimentation, or texture alteration	Appearance: Homogeneous<br>Color: amber yellow<br>Smell: Aromatic, specific<br>No signs of phase separation, sedimentation, or texture alteration	Appearance: Homogeneous<br>Color: pale yellow<br>Smell: Aromatic, specific<br>No signs of phase separation, sedimentation, or texture alteration
pH - after 30 days, room temperature	5.29 ± 0.02	5.37 ± 0.03	5.25 ± 0.03	5.30 ± 0.05	5.33 ± 0.04
Organoleptic evaluation - after 60 days, room temperature	Appearance: Homogeneous<br>Color: yellowish<br>Smell: Aromatic, specific<br>No signs of phase separation, sedimentation, or texture alteration	Appearance: Homogeneous<br>Color: light yellow<br>Smell: Aromatic, specific<br>No signs of phase separation, sedimentation, or texture alteration,	Appearance: Homogeneous<br>Color: light yellow<br>Smell: Aromatic, specific<br>No signs of phase separation, sedimentation, or texture alteration	Appearance: Homogeneous<br>Color: burnt yellow<br>Smell: Aromatic, specific<br>No signs of phase separation, sedimentation, or texture alteration	Appearance: Homogeneous<br>Color: mustard yellow<br>Smell: Aromatic, specific<br>No signs of phase separation, sedimentation, or texture alteration
pH - after 60 days, room temperature	5.29 ± 0.09	5.38 ± 0.06	5.26 ± 0.09	5.31 ± 0.06	5.34 ± 0.07

Table S14. TPA profile of the first gel-based cosmetic formulation enriched with grape pomace (Feteasca Neagra and Merlot varieties) and Japanese knotweed (*Polygonum cuspidatum*) extracts (24 h).

Characteristics	CBG1-M-FN/PcF	CBG1-M-FN/PcR	CBG1-M-FN	CBG1-PcF	CBG1-PcR
Firmness (hardness)	0.553 ± 0.017 N	0.401 ± 0.012 N	0.466 ± 0.014 N	0.397 ± 0.012 N	0.364 ± 0.011 N
Cohesiveness	0.662 ± 0.020	0.637 ± 0.019	0.625 ± 0.019	0.625 ± 0.019	0.608 ± 0.018
Springiness	0.980 ± 0.029	0.838 ± 0.025	0.862 ± 0.026	0.829 ± 0.025	0.735 ± 0.022

Table S15. TPA profile of the second gel-based cosmetic formulation enriched with grape pomace (Feteasca Neagra and Merlot varieties) and Japanese knotweed (*Polygonum cuspidatum*) extracts (24 h).

Characteristics	CBG1.25-M-FN/PcF	CBG1.25-M-FN/PcR	CBG1.25-M-FN	CBG1.25-PcF	CBG1.25-PcR
Firmness (hardness)	0.392 ± 0.012 N	0.368 ± 0.011 N	0.542 ± 0.016 N	0.395 ± 0.012 N	0.553 ± 0.017 N
Cohesiveness	0.661 ± 0.020	0.665 ± 0.020	0.605 ± 0.018	0.641 ± 0.019	0.597 ± 0.018
Springiness	0.806 ± 0.024	0.811 ± 0.024	0.717 ± 0.022	0.784 ± 0.024	0.869 ± 0.026

Table S16. TPA profile of the first gel-based cosmetic formulation enriched with grape pomace (Feteasca Neagra and Merlot varieties) and Japanese knotweed (*Polygonum cuspidatum*) extracts (30 days).

Characteristics	CBG1-M-FN/PcF	CBG1-M-FN/PcR	CBG1-M-FN	CBG1-PcF	CBG1-PcR
Firmness (hardness)	0.554 ± 0.017 N	0.411 ± 0.012 N	0.468 ± 0.014 N	0.399 ± 0.012 N	0.368 ± 0.011 N
Cohesiveness	0.665 ± 0.020	0.639 ± 0.019	0.629 ± 0.019	0.627 ± 0.019	0.618 ± 0.019
Springiness	0.981 ± 0.029	0.839 ± 0.025	0.864 ± 0.026	0.832 ± 0.025	0.739 ± 0.022

Table S17. TPA profile of the second gel-based cosmetic formulation enriched with grape pomace (Feteasca Neagra and Merlot varieties) and Japanese knotweed (*Polygonum cuspidatum*) extracts (30 days).

Characteristics	CBG1.25-M-FN/PcF	CBG1.25-M-FN/PcR	CBG1.25-M-FN	CBG1.25-PcF	CBG1.25-PcR
Firmness (hardness)	0.421 ± 0.013 N	0.370 ± 0.011 N	0.549 ± 0.016 N	0.411 ± 0.012 N	0.543 ± 0.016 N
Cohesiveness	0.669 ± 0.020	0.679 ± 0.020	0.597 ± 0.018	0.648 ± 0.019	0.598 ± 0.018
Springiness	0.876 ± 0.026	0.834 ± 0.025	0.727 ± 0.022	0.799 ± 0.024	0.870 ± 0.026

Table S18. TPA profile of the first gel-based cosmetic formulation enriched with grape pomace (Feteasca Neagra and Merlot varieties) and Japanese knotweed (*Polygonum cuspidatum*) extracts (60 days)

**Characteristics**	**CBG1-M-FN/PcF**	**CBG1-M-FN/PcR**	**CBG1-M-FN**	**CBG1-PcF**	**CBG1-PcR**
Firmness (hardness)	0.553 ± 0.017 N	0.434 ± 0.013 N	0.579 ± 0.017 N	0.380 ± 0.011 N	0.428 ± 0.013 N
Cohesiveness	0.690 ± 0.021	0.673 ± 0.020	0.641 ± 0.019	0.654 ± 0.020	0.691 ± 0.021
Springiness	0.828 ± 0.015	0.857 ± 0.026	0.766 ± 0.023	0.817 ± 0.025	0.870 ± 0.026

Table S19. TPA profile of the second gel-based cosmetic formulation enriched with grape pomace (Feteasca Neagra and Merlot varieties) and Japanese knotweed (*Polygonum cuspidatum*) extracts (60 days).

**Characteristics**	**CBG1.25-M-FN/PcF**	**CBG1.25-M-FN/PcR**	**CBG1.25-M-FN**	**CBG1.25-PcF**	**CBG1.25-PcR**
Firmness (hardness)	0.486 ± 0.015 N	0.462 ± 0.014 N	0.648 ± 0.019 N	0.436 ± 0.013 N	0.533 ± 0.016 N
Cohesiveness	0.685 ± 0.021	0.653 ± 0.020	0.592 ± 0.018	0.663 ± 0.020	0.619 ± 0.019
Springiness	0.886 ± 0.027	0.872 ± 0.026	0.774 ± 0.023	0.775 ± 0.023	0.851 ± 0.020
